# Siglec-9 Restrains Antibody-Dependent Natural Killer Cell Cytotoxicity against SARS-CoV-2

**DOI:** 10.1128/mbio.03393-22

**Published:** 2023-02-02

**Authors:** Pratima Saini, Opeyemi S. Adeniji, Devivasha Bordoloi, Jennifer Kinslow, Jeff Martinson, Danielle M. Parent, Kai Ying Hong, Jane Koshy, Abhijeet J. Kulkarni, Netanel F. Zilberstein, Robert A. Balk, James N. Moy, Leila B. Giron, Russell P. Tracy, Ali Keshavarzian, Kar Muthumani, Alan Landay, David B. Weiner, Mohamed Abdel-Mohsen

**Affiliations:** a The Wistar Institute, Philadelphia, Pennsylvania, USA; b Rush University, Chicago, Illinois, USA; c University of Vermont, Burlington, Vermont, USA; Dartmouth College

**Keywords:** SARS-CoV-2, COVID-19, natural killer cells, Siglec-7, Siglec-9, antibody-dependent cell cytotoxicity

## Abstract

Severe acute respiratory syndrome coronavirus 2 (SARS-CoV-2) infection alters the immunological profiles of natural killer (NK) cells. However, whether NK antiviral functions are impaired during severe coronavirus disease 2019 (COVID-19) and what host factors modulate these functions remain unclear. We found that NK cells from hospitalized COVID-19 patients degranulate less against SARS-CoV-2 antigen-expressing cells (in direct cytolytic and antibody-dependent cell cytotoxicity [ADCC] assays) than NK cells from mild COVID-19 patients or negative controls. The lower NK degranulation was associated with higher plasma levels of SARS-CoV-2 nucleocapsid antigen. Phenotypic and functional analyses showed that NK cells expressing the glyco-immune checkpoint Siglec-9 elicited higher ADCC than Siglec-9^–^ NK cells. Consistently, Siglec-9^+^ NK cells exhibit an activated and mature phenotype with higher expression of CD16 (FcγRIII; mediator of ADCC), CD57 (maturation marker), and NKG2C (activating receptor), along with lower expression of the inhibitory receptor NKG2A, than Siglec-9^–^ CD56^dim^ NK cells. These data are consistent with the concept that the NK cell subpopulation expressing Siglec-9 is highly activated and cytotoxic. However, the Siglec-9 molecule itself is an inhibitory receptor that restrains NK cytotoxicity during cancer and other viral infections. Indeed, blocking Siglec-9 significantly enhanced the ADCC-mediated NK degranulation and lysis of SARS-CoV-2-antigen-positive target cells. These data support a model in which the Siglec-9^+^ CD56^dim^ NK subpopulation is cytotoxic even while it is restrained by the inhibitory effects of Siglec-9. Alleviating the Siglec-9-mediated restriction on NK cytotoxicity may further improve NK immune surveillance and presents an opportunity to develop novel immunotherapeutic tools against SARS-CoV-2 infected cells.

## INTRODUCTION

Most individuals infected with severe acute respiratory syndrome coronavirus 2 (SARS-CoV-2) experience mild symptoms; however, many require hospitalization (1, 2). The mechanisms underlying coronavirus disease 2019 (COVID-19) severity are likely multifactorial, and disruption of immunological functions could be one of these mechanisms. Indeed, severe COVID-19 has been associated with alterations to the profiles of several immune cells (3, 4), including natural killer (NK) cells (5–7). NK cells are effector innate immune cells that play a central role in antiviral immunity through direct cytotoxicity and/or antibody-dependent cell cytotoxicity (ADCC) (8). However, whether severe COVID-19 impairs the anti-SARS-CoV-2 functions of NK cells and what host factors modulate these functions remain unclear.

The cytotoxic potential of NK cells is determined by the balance of opposing signals resulting from multiple activating (such as NKG2C) and inhibitory (such as NKG2A) receptors expressed on the surface of these cells (9–11). Among the inhibitory receptors, NK cells express two that belong to a family of emerging glyco-immune checkpoints called Siglecs: Siglec-7 and Siglec-9 (12, 13). Siglecs are sialic-acid-binding, immunoglobulin-like lectins that inhibit immune functions by interacting with sialoglycans (sialic acid-containing glycomic structures) on target cells and signaling through intracellular immunoreceptor tyrosine-based inhibitory motifs (ITIMs) (14). In cancer, Siglec-sialoglycan interactions help tumor cells to evade NK immune surveillance (15–17). Recently, these interactions have been suggested to also help hepatitis B virus (HBV)- and HIV-infected cells to evade NK immune surveillance (13, 18). Despite a growing appreciation of Siglecs as glyco-immune negative checkpoints during cancer and viral infections, their role in helping SARS-CoV-2 evade immune surveillance has never been examined.

In this study, we first examined the impact of severe COVID-19 on the degranulation/cytokine production of NK cells (in direct cytotoxicity and ADCC assays). We found that NK cells from hospitalized COVID-19 patients degranulate less against SARS-CoV-2 Spike-expressing target cells than NK cells from mild COVID-19 patients or SARS-CoV-2 negative controls. This lower NK function against SARS-CoV-2 Spike-expressing target cells was correlated with higher viral antigen loads in plasma, suggesting that the impaired NK function may be clinically relevant. Having found that NK degranulation against SARS-CoV-2 Spike-expressing target cells is impaired during severe disease, we then sought to understand some of the molecular mechanisms involved in NK degranulation and cytotoxicity against SARS-CoV-2. We focused on determining the role of Siglecs in modulating the anti-SARS-CoV-2 NK cytotoxicity. We found that the NK subpopulation expressing the glyco-immune checkpoint Siglec-9 elicits higher ADCC against SARS-CoV-2 than Siglec-9^–^ NK cells. By performing a detailed *in vivo* phenotypic analysis, we found that the Siglec-9^+^ NK subpopulation exhibits a more activated and mature phenotype than the Siglec-9^–^ NK subpopulation (regardless of disease status); this phenotype is consistent with the high cytotoxic activity of this subpopulation. However, we also found that the expression of the inhibitory molecule Siglec-9 restrains the cytotoxic activity of this otherwise highly activated subpopulation of NK cells; blocking Siglec-9 interactions using a blocking antibody significantly improved the ability of NK cells to elicit ADCC against SARS-CoV-2.

## RESULTS

### NK cells from hospitalized COVID-19 patients degranulate less than NK cells from mild COVID-19 patients or uninfected controls against SARS-CoV-2 Spike-expressing target cells.

**FIG 1:**
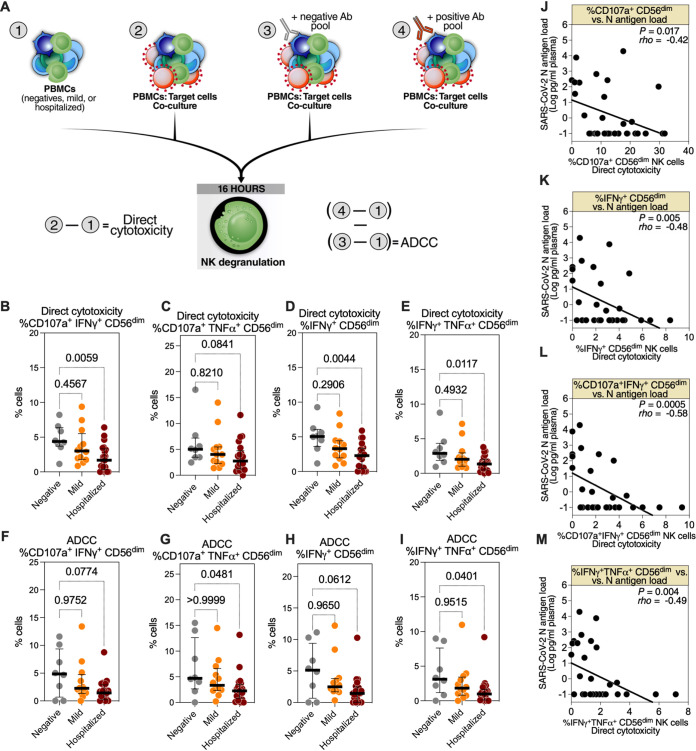
Hospitalized coronavirus disease 2019 (COVID-19) is associated with reduced CD56^dim^ natural killer (NK) cell degranulation against severe acute respiratory syndrome coronavirus 2 (SARS-CoV-2) Spike-expressing target cells. (A) Schematic overview of the experiments to evaluate the direct cytotoxicity- and antibody-dependent cell cytotoxicity (ADCC)-mediated degranulation of CD56^dim^ NK cells during different severities of COVID-19. To examine direct cytotoxicity-mediated degranulation, peripheral blood mononuclear cells (PBMCs) from each donor, from three COVID-19 status groups (*n* = 8 SARS-CoV-2 negative, *n* = 12 mild COVID-19, and *n* = 21 hospitalized COVID-19), were cocultured (2) or not (1) with SARS-CoV-2 Spike-expressing 293T target cells. To examine ADCC-mediated degranulation, identical cocultures were performed in the presence of the negative (3) or positive (4) antibody pool. Direct cytotoxicity was assessed by subtracting (1) from (2). 10:1 effector:target [E:T] ratio. ADCC was assessed by subtracting the results of subtracting (1) from (3) from the results of subtracting (1) from (4). (B to E) Direct cytotoxicity-mediated degranulation and cytokine production of the CD56^dim^ NK population was assessed as the percentage of (B) CD107a^+^ interferon γ (IFN-γ)^+^, (C) CD107a^+^ tumor necrosis factor α (TNF-α)^+^, (D) IFN-γ^+^, and (E) IFN-γ^+^ TNF-α^+^ cells. Medians and interquartile ranges (IQR) are displayed. Kruskal-Wallis tests with Dunn’s multiple comparisons correction were used for statistical analyses. (F to I) ADCC-mediated degranulation and cytokine production of the CD56^dim^ NK population was assessed as the percentage of (F) CD107a^+^ IFN-γ^+^, (G) CD107a^+^ TNF-α^+^, (H) IFN-γ^+^, or (I) IFN-γ^+^ TNF-α^+^ cells. Median and IQR are shown. Kruskal-Wallis tests with Dunn’s multiple-comparisons correction were used for statistical analyses. (J to M) Spearman’s rank-order correlations between plasma N-antigen load and the percentage of CD56^dim^ NK cells expressing (J) CD107a^+^, (K) IFN-γ^+^, (L) CD107a^+^ IFN-γ^+^, and (M) IFN-γ^+^ TNF-α^+^ during direct cytotoxicity assays; only samples from COVID-19-positive donors were used (*n* = 33).

To examine whether severe COVID-19 impairs the anti-SARS-CoV-2 functions of NK cells, we collected peripheral blood mononuclear cells (PBMCs) and plasma from 79 individuals with three COVID-19 disease states: (i) SARS-CoV-2 negative control (negative; *n* = 12), (ii) COVID-19 outpatients (mild; *n* = 26), and (iii) COVID-19 inpatients (hospitalized; *n* = 41) (Table S1). First, using samples from a subset of these individuals with sufficient PBMCs (*n* = 8 negative, *n* = 12 mild, and *n* = 21 hospitalized), we assessed the anti-SARS-CoV-2-specific direct cytolytic and ADCC activities of NK cells. NK cells may be polyfunctional, directly lysing target cells by releasing cytolytic granules and secreting cytokines and chemokines such as interferon γ (IFN-γ) and tumor necrosis factor α (TNF-α) (19). The polyfunctionality of NK cells has been associated with enhanced antiviral immune responses (20). Therefore, we assessed direct cytotoxicity and ADCC by both NK degranulation (expression of CD107a) and cytokine production (the expression of IFN-γ and TNF-α) against SARS-CoV-2 Spike-expressing 293T target cells measured by flow cytometry (gating strategy is described in Fig. S1A). Direct cytotoxicity was calculated by subtracting the background NK degranulation/cytokine production of PBMCs cultured alone from the NK degranulation/cytokine production of PBMCs cocultured with target cells (Fig. 1A, left). To assess ADCC, we isolated bulk IgGs from the plasma of the study participants. The IgGs from the SARS-CoV-2-negative donors were pooled to create a negative antibody pool, and the IgGs from the SARS-CoV-2-positive donors were pooled to create a positive antibody pool. We pooled IgGs to ensure that the quantitative and qualitative features of the antibodies used in the ADCC assays were constant. Having constant levels of SARS-CoV-2-specific antibodies would allow examination of the ADCC capacity of NK cells from different donors without the potential confounding effects of different levels or qualities of SARS-CoV-2-specific antibodies. NK degranulation/cytokine production by ADCC was measured by coculturing PBMCs and target cells in the presence of the negative or positive antibody pools. ADCC was then assessed by subtracting the percentage of NK degranulation/cytokine production of the coculture with the negative antibody pool from that of the coculture with the positive antibody pool (after subtracting the background NK degranulation/cytokine production) (Fig. 1A, right).

**FIG 2:**
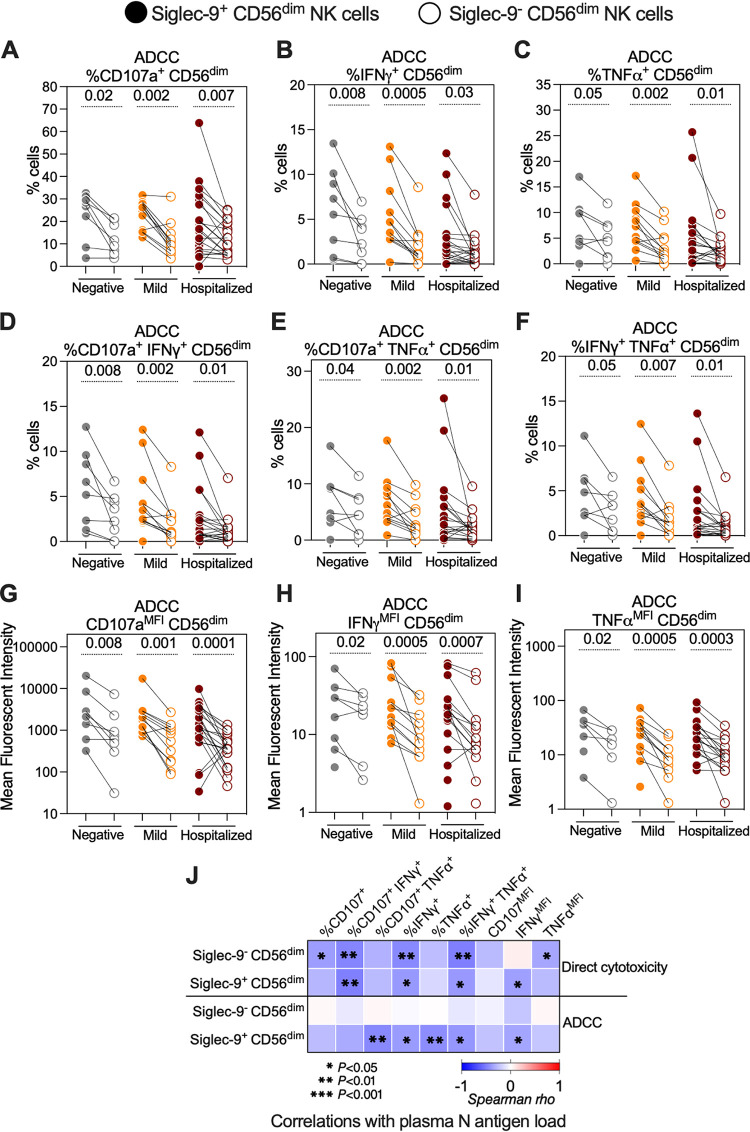
The Siglec-9^+^ CD56^dim^ NK subpopulation exhibits a higher SARS-CoV-2 specific ADCC than the Siglec-9^–^ CD56^dim^ NK subpopulation. (A to I) ADCC-mediated degranulation and/or cytokine production of the Siglec-9^+^ and Siglec-9^–^ CD56^dim^ NK cells within each COVID-19 status group as assessed by the percentage of cells expressing (A) CD107a, (B) IFN-γ, (C) TNF-α, (D) CD107a and IFN-γ, (E) CD107a and TNF-α, and (F) IFN-γ and TNF-α. The mean fluorescence intensity (MFI) was also evaluated for (G) CD107a, (H) IFN-γ, and (I) TNF-α. Wilcoxon’s signed-rank tests were used to compare the Siglec-9^+^ and Siglec-9^–^ CD56^dim^ NK cells within each COVID-19 status group. (J) Spearman’s correlation heat-map showing associations between direct cytotoxicity and ADCC-mediated NK degranulation of the Siglec-9^+^ and Siglec-9^–^ CD56^dim^ NK cells and plasma N-antigen load. Square color represents correlation strength, with blue representing negative correlations and red representing positive correlations; only samples from COVID-19-positive donors were used (*n* = 33).

**FIG 3:**
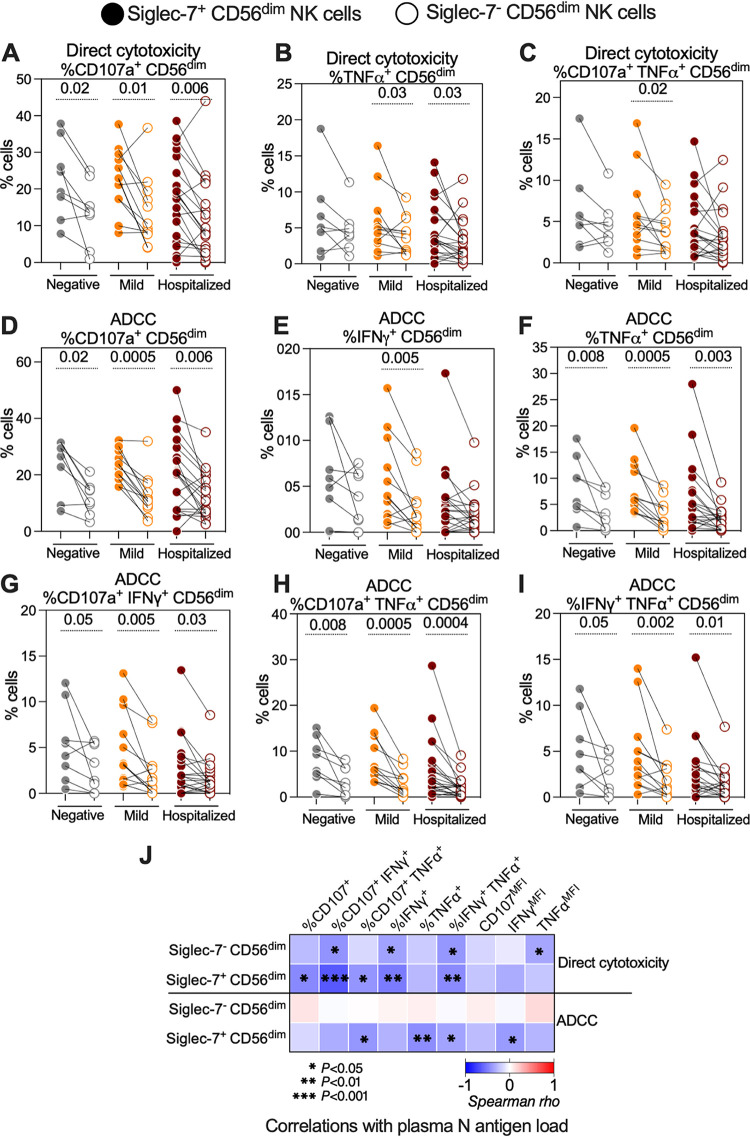
The Siglec-7^+^ CD56^dim^ NK subpopulation exhibits higher SARS-CoV-2-specific direct cytolytic and ADCC activity than does the Siglec-7^–^ CD56^dim^ NK subpopulation. (A to C) Direct cytotoxicity-mediated degranulation and/or cytokine production of the Siglec-7^+^ and Siglec-7^–^ CD56^dim^ NK cells within each COVID-19 status group as assessed by the percentage of cells expressing (A) CD107a, (B) TNF-α, and (C) CD107a and TNF-α. (D to I) ADCC-mediated degranulation and/or cytokine production of the Siglec-7^+^ and Siglec-7^–^ CD56^dim^ NK cells within each COVID-19 status group as assessed by the percentage of cells expressing (D) CD107a, (E) IFN-γ, (F) TNF-α, (G) CD107a and IFN-γ, (H) CD107a and TNF-α, or (I) IFN-γ and TNF-α. Wilcoxon’s signed-rank tests were used to compare the Siglec-7^+^ and Siglec-7^–^ CD56^dim^ NK cells within each disease group. (J) Spearman’s correlation heat-map showing associations between direct cytotoxicity- and ADCC-mediated NK degranulation of the Siglec-7^+^ and Siglec-7^–^ CD56^dim^ NK cells and plasma N-antigen load. Square color represents correlation strength, with blue representing negative correlations and red representing positive correlations; only samples from COVID-19-positive donors were used (*n* = 33).

10.1128/mbio.03393-22.1 FIG S1 Mean fluorescence intensity of CD107a, IFN-γ, and TNF-α in CD56^dim^ NK cells after co-culturing with SARS-CoV-2 Spike-expressing 293T cells. (A) Gating strategy for experiments shown in Fig. 1, 2, 3, Fig. 5F to H, and Fig. 6A to E. (B to G) Direct cytotoxicity and antibody-dependent cell cytotoxicity (ADCC) were calculated, as described in Fig. 1, within each COVID-19 status group (*n* = 8 SARS-CoV-2-negative, *n* = 12 mild COVID-19, and *n* = 21 hospitalized COVID-19). The mean fluorescence intensity (MFI) was evaluated for direct cytotoxicity, (B) CD107a, (C) interferon γ (IFN-γ), and (D) tumor necrosis factor α (TNF-α). MFI was also evaluated for ADCC, (E) CD107a, (F) IFN-γ, and (G) TNF-α. Median and interquartile range (IQR) are displayed. Kruskal-Wallis tests with Dunn’s multiple-comparisons correction were used for statistical analyses. Download FIG S1, PDF file, 0.9 MB. Copyright © 2023 Saini et al. 2023 Saini et al. https://creativecommons.org/licenses/by/4.0/ This content is distributed under the terms of the Creative Commons Attribution 4.0 International license.

10.1128/mbio.03393-22.10 TABLE S1 Demographic and clinical characteristics of the study cohort. Download Table S1, XLSX file, 0.01 MB. Copyright © 2023 Saini et al. 2023 Saini et al. https://creativecommons.org/licenses/by/4.0/ This content is distributed under the terms of the Creative Commons Attribution 4.0 International license.

First, we found that the direct cytolytic-mediated degranulation/cytokine production (Fig. 1B to E, Fig. S1B to D) and ADCC-mediated degranulation (Fig. 1F to I, Fig. S1E to G) against SARS-CoV-2 Spike-expressing cells of CD56^dim^ NK cells from hospitalized COVID-19 patients were lower than those of the NK cells from the mild group or the SARS-CoV-2 negative controls. In a sub-analysis, we found that the cytotoxic activities of NK cells from the female participants were higher than those from the male participants, suggesting a sex-dependent difference in NK activity against SARS-CoV-2 Spike-expressing targets (Fig. S2). Next, we examined whether the reduced degranulation/cytokine production observed from the NK cells of hospitalized COVID-19 donors results in decreased lysis of SARS-CoV-2 Spike-expressing target cells. For these experiments, we used PBMCs from hospitalized or mild COVID-19 patients as effector cells and SARS-CoV-2 S CHO-K1 cells as target cells. The SARS-CoV-2 S CHO-K1 cells stably express the SARS-CoV-2 Spike (S) protein and a HaloTag-HiBiT protein; when these cells are lysed by ADCC, the intracellular HaloTag-HiBiT protein interacts with an extracellular detection reagent to generate a luminescence signal that can be quantified to measure target cell lysis. As shown in Fig. S3A, PBMCs from hospitalized COVID-19 donors exhibited lower ADCC than those from mild COVID-19 donors, consistent with the NK degranulation/cytokine production data. Finally, we examined the cytotoxicity of NK cells from the three COVID-19 disease states against cells not expressing SARS-CoV-2 Spike protein (K562 cells). We found that NK cells from hospitalized COVID-19 patients degranulate less against K562 cells than NK cells from the mild group or the SARS-CoV-2 negative controls (Fig. S3B to F). These data suggest that these NK cells from hospitalized COVID-19 patients have a general impairment of cytotoxic activity.

10.1128/mbio.03393-22.2 FIG S2 Sex-dependent differences in cytolytic activity for NK cells against SARS-CoV-2 Spike-expressing 293 T cells. (A and B) The direct cytolytic and ADCC activities of CD56^dim^ NK cells from SARS-CoV-2 negative controls (*n* = 8; grey dots), mild COVID-19 donors (*n* = 12; orange dots), and hospitalized COVID-19 donors (*n* = 21; maroon dots) were compared based on sex (female; *n* = 18, and male; *n* = 23). Direct cytotoxicity (A) and ADCC (B) were calculated as described in Fig. 1. Direct cytotoxicity and ADCC were measured as the percentage of cells expressing CD107a^+^, IFN-γ^+^, TNF-α^+^, CD107a^+^ IFN-γ^+^, CD107a^+^ TNF-α^+^, and IFN-γ^+^ TNF-α^+^, and the mean fluorescence intensity (MFI) of CD107a, IFN-γ, and TNF-α. Median and IQR are displayed. Mann-Whitney U tests were used for statistical analyses. Download FIG S2, PDF file, 0.8 MB. Copyright © 2023 Saini et al. 2023 Saini et al. https://creativecommons.org/licenses/by/4.0/ This content is distributed under the terms of the Creative Commons Attribution 4.0 International license.

10.1128/mbio.03393-22.3 FIG S3 Hospitalized COVID-19 is associated with reduced NK cytotoxicity. (A) The ADCC-mediated lysis of SARS-CoV-2 Spike-expressing CHO target cells by peripheral blood mononuclear cells (PBMCs) from mild (*n* = 8) and hospitalized COVID-19 donors (*n* = 9) was examined. CHO-K1 target cells were incubated with either the positive or negative SARS-CoV-2 antibody pools for 15 min. After 15 min, the cells were co-cultured at a 10:1 effector:target (E:T) ratio for 5 h. The SARS-CoV-2 S CHO-K1 cells stably express the SARS-CoV-2 Spike (S) protein and a HaloTag-HiBiT protein. When the target cells are lysed by ADCC, the intracellular HaloTag-HiBiT protein interacts with the extracellular detection reagent to generate a luminescence signal that can quantitatively measure the degree of target cell lysis. Luminescence values of each donor for the positive pool were subtracted by the values obtained from the respective negative pool to obtain the target cell lysis values. Median and IQR are shown. Mann-Whitney U test was used for statistical analyses. (B to F) Hospitalized COVID-19 is associated with reduced CD56^dim^ NK cells degranulation against K562 target cells. PBMCs from three COVID-19 status groups (*n* = 11 SARS-CoV-2-negative, *n* = 9 mild COVID-19, and *n* = 16 hospitalized COVID-19) were co-cultured at a 5:1 (E:T) ratio with K562. Direct cytotoxicity was assessed by subtracting the degranulation/cytokine production of PBMCs culture alone from that of PBMCs co-cultured with target cells. Direct cytotoxicity-mediated degranulation and cytokine production of the CD56^dim^ NK population against K562 target cells was assessed as the percentage of (B) CD107a^+^, (C) IFN-γ^+^, (D) TNF-α^+^, (E) CD107a^+^ IFN-γ^+^, and (F) CD107a^+^ TNF-α^+^ cells. Median and IQR are shown. Kruskal-Wallis tests with Dunn’s multiple-comparisons correction were used for statistical analyses. Download FIG S3, PDF file, 0.5 MB. Copyright © 2023 Saini et al. 2023 Saini et al. https://creativecommons.org/licenses/by/4.0/ This content is distributed under the terms of the Creative Commons Attribution 4.0 International license.

We next examined whether the NK degranulation measured *ex vivo* correlated with markers of disease severity *in vivo*. Plasma SARS-CoV-2 plasma nucleocapsid (N) antigen load correlates with disease severity (21–23). Therefore, we measured the N-antigen load in the plasma samples using an ultrasensitive Simoa SARS-CoV-2 N-protein assay and correlated it with NK direct cytolytic and ADCC activities. As shown in Fig. 1J to M, NK degranulation was inversely correlated with SARS-CoV-2 N-antigen load. Together, these data suggest that NK degranulation against SARS-CoV-2 is compromised during severe COVID-19. Therefore, developing strategies to enhance anti-SARS-CoV-2 NK cytotoxicity may improve disease outcomes. However, developing such strategies requires a better understanding of the factors that determine NK functions against SARS-CoV-2.

### Siglec-9^+^ CD56^dim^ NK cells exhibit higher antibody-mediated cytotoxicity against SARS-CoV-2-Spike-expressing target cells than do Siglec-9^–^ CD56^dim^ NK cells.

Having found that NK degranulation against SARS-CoV-2 is impaired during severe disease, we then sought to understand some of the molecular mechanisms involved in NK cytotoxicity against SARS-CoV-2. We focused on determining whether the Siglecs expressed on NK cells, Siglec-9 and Siglec-7, play a role in establishing either the direct cytolytic activity or the ADCC activity of NK cells against SARS-CoV-2 Spike-expressing target cells. First, we used data from the experiments shown in Fig. 1 after gating on either Siglec-9^+^ CD56^dim^ NK cells or Siglec-9^–^ CD56^dim^ NK cells. We did not observe a difference in the direct cytolytic activity of these two subpopulations; however, the Siglec-9^+^ CD56^dim^ NK cells exhibited significantly higher ADCC against target cells than the Siglec-9^–^ CD56^dim^ NK cells, irrespective of disease state (Fig. 2A to I). This was consistently observed when we evaluated the percentage of cells expressing CD107a, IFN-γ, and TNF-α (Fig. 2A to C) or co-expressing CD107a and IFN-γ, CD107a and TNF-α, or IFN-γ and TNF-α (Fig. 2D to F) in each of the two subpopulations. It was also consistent when we evaluated the mean fluorescence intensities (MFI) of CD107a, IFN-γ, and TNF-α for each subpopulation (Fig. 2G to I). Next, we examined the associations between the direct cytolytic or ADCC activities of the Siglec-9^+^ CD56^dim^ and the Siglec-9^–^ CD56^dim^ subpopulations, measured *ex vivo*, and the plasma N-antigen load, measured *in vivo* (Fig. 2J). The direct cytolytic activity of both subpopulations correlated negatively with the plasma N-antigen load, indicating that the Siglec-9^+^ NK subpopulation probably does not play a unique role in anti-SARS-CoV-2 direct cytotoxicity. However, the ADCC ability of the Siglec-9^+^, but not the Siglec-9^–^, CD56^dim^ NK cells correlated negatively with the plasma N-antigen load (Fig. 2J). These data suggest that Siglec-9^+^ CD56^dim^ NK cells are a subpopulation of NK cells with potentially high ADCC activity.

### Siglec-7^+^ CD56^dim^ NK cells exhibit higher direct cytotoxicity and ADCC toward SARS-CoV-2-Spike-expressing target cells than do Siglec-7^–^ CD56^dim^ NK cells.

We next examined the direct cytolytic and ADCC activities of the Siglec-7^+^ CD56^dim^ NK and Siglec-7^–^ CD56^dim^ NK cells (Fig. 3). Unlike Siglec-9^+^ CD56^dim^ NK cells, Siglec-7^+^ CD56^dim^ NK cells exhibited higher direct cytolytic activity against SARS-CoV-2 Spike-expressing target cells than their Siglec-7^–^ counterparts, as estimated by the percentage of cells expressing CD107a, TNF-α, and both CD107a and TNF-α (Fig. 3A to C). In addition, the Siglec-7^+^ CD56^dim^ NK cells exhibited higher SARS-CoV-2-specific ADCC than did the Siglec-7^–^ CD56^dim^ NK cells, as estimated by the percentage of cells expressing CD107a, IFN-γ, and TNF-α (Fig. 3D to F) or co-expressing CD107a and IFN-γ, CD107a and TNF-α, or IFN-γ and TNF-α (Fig. 3G to I). These data were also consistent when examining the MFI of CD107a, IFN-γ, and TNF-α (Fig. S4). These cytolytic activities correlated more strongly with lower plasma N-antigen loads compared with the activities of the Siglec-7^–^ CD56^dim^ NK subpopulation (Fig. 3J), a result that is consistent with the high direct cytolytic and ADCC activity of the Siglec-7^+^ CD56^dim^ NK subpopulation. The Siglec-7^–^ NK subpopulation has been described as a dysfunctional NK subpopulation during other viral infections, such as HIV infection (24–26). Our data suggest that this subpopulation is also dysfunctional during SARS-CoV-2 infection.

10.1128/mbio.03393-22.4 FIG S4 The Siglec7^+^ CD56^dim^ NK subpopulation exhibits higher SARS-CoV-2-specific ADCC activities than the Siglec-7^–^ CD56^dim^ NK subpopulation. (A to C) ADCC-mediated degranulation of the Siglec-7^+^ and Siglec-7^–^ CD56^dim^ NK cells within each COVID-19 status group (*n* = 8 SARS-CoV-2 negative; *n* = 12 mild COVID-19, and *n* = 21 hospitalized COVID-19) as assessed by the MFI. MFI was also evaluated for (A) CD107a, (B) IFN-γ, and (C) TNF-α. Wilcoxon’s signed-rank tests were used to compare the Siglec-9^+^ and Siglec-9^–^ CD56^dim^ NK cells within each disease group. Download FIG S4, PDF file, 0.2 MB. Copyright © 2023 Saini et al. 2023 Saini et al. https://creativecommons.org/licenses/by/4.0/ This content is distributed under the terms of the Creative Commons Attribution 4.0 International license.

### Siglec-9^+^ CD56^dim^ NK cells exhibit an activated and mature phenotype *in vivo*.

Our data so far suggest that the NK subpopulations which express Siglec-9 or Siglec-7 exhibit higher cytotoxicity than their Siglec-negative counterparts. We therefore examined whether this high cytotoxicity is due to the differential expression of other activating or inhibitory receptors on the surface of these NK cells. Therefore, we evaluated the expression of several activating and inhibitory receptors on NK cells from the entire cohort (*n* = 79). In particular, we measured the expression of CD16 (FcγRIII; mediator of ADCC), CD57 (maturation marker), NKG2C (activating receptor), and NKG2A (inhibitory receptor) on Siglec-9^+^, Siglec-9^–^, Siglec-7^+^, and Siglec-7^–^ CD56^dim^ NK cells (gating strategy is described in Fig. S5). Given that Siglec-9 is expressed only on a subset of CD56^dim^ NK cells, we used quantitative PCR (qPCR) to validate the specificity of the Siglec-9 antibody (Ab) (clone K8; Biolegend) in identifying NK subpopulations with high levels of Siglec-9 transcription. The data in Fig. S6 show that this Ab identifies cells with higher levels of Siglec-9 transcription compared to Siglec-9-negative NK cells. In a sub-analysis, we also found that Siglec-7 and Siglec-9 expression on CD56^dim^ NK cells is sex-dependent. In particular, CD56^dim^ NK cells from the female participants had higher levels of Siglec-9^+^, Siglec-7^+^, Siglec-9^–^ Siglec-7^+^, and Siglec-9^+^ Siglec-7^+^ and lower levels of Siglec-9^–^ Siglec7^–^ and Siglec-9^+^ Siglec-7^–^ than cells from the male participants (Fig. S7).

**FIG 4:**
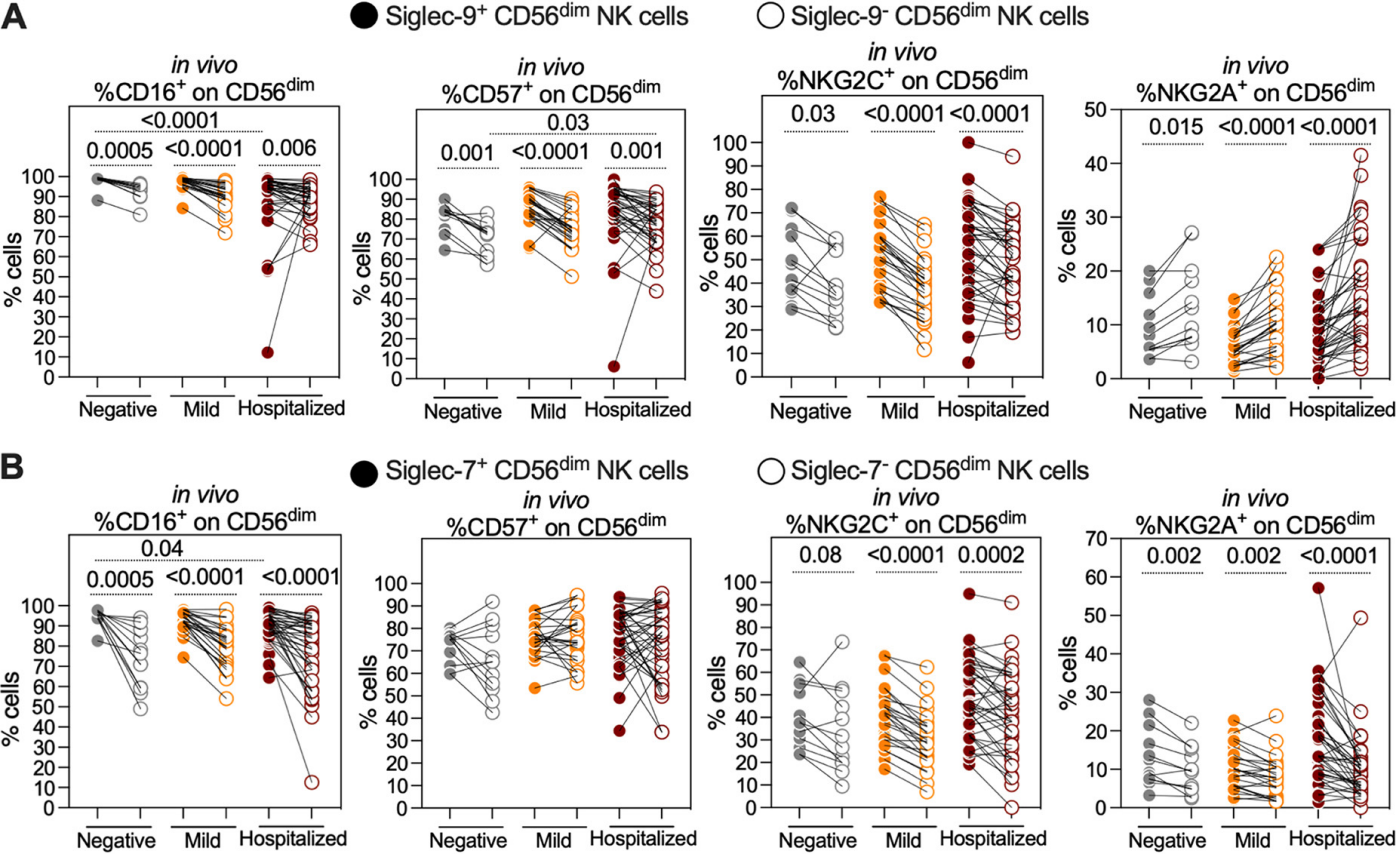
Siglec-9 marks activated and mature CD56^dim^ NK subpopulation *in vivo*. (A) Comparisons of the expression of CD16 (FcγRIII; mediator of ADCC), CD57 (maturation marker), NKG2C (activating receptor), and NKG2A (inhibitory receptor) on Siglec-9^+^ and Siglec-9^–^ CD56^dim^ NK cells obtained from 79 individuals with three COVID-19 disease states (negative; *n* = 12, mild; *n* = 26, and hospitalized; *n* = 41). Siglec-9^+^ cells exhibit higher levels of CD16, CD57, NKG2C, and lower levels of NKG2A compared to Siglec-9^–^ cells. Wilcoxon’s signed-rank tests were used to compare the Siglec-9^+^ and Siglec-9^–^ CD56^dim^ NK cells within each COVID-19 disease state group. Mann-Whitney U tests were used to compare disease state groups. (B) Comparisons of CD16, CD57, NKG2C, and NKG2A expression on Siglec-7^+^ and Siglec-7^–^ CD56^dim^ NK cells. Siglec-7^+^ cells exhibited higher levels of CD16, NKG2C, and NKG2A than Siglec-7^–^ cells. No differences were observed in CD57 expression between the Siglec-7^+^ and Siglec-7^–^ cells. As in panel A, Wilcoxon’s signed-rank tests were used to compare the Siglec-7^+^ and Siglec-7^–^ CD56^dim^ NK cells within each COVID-19 status group, and Mann-Whitney U tests were used to compare the status groups.

**FIG 5:**
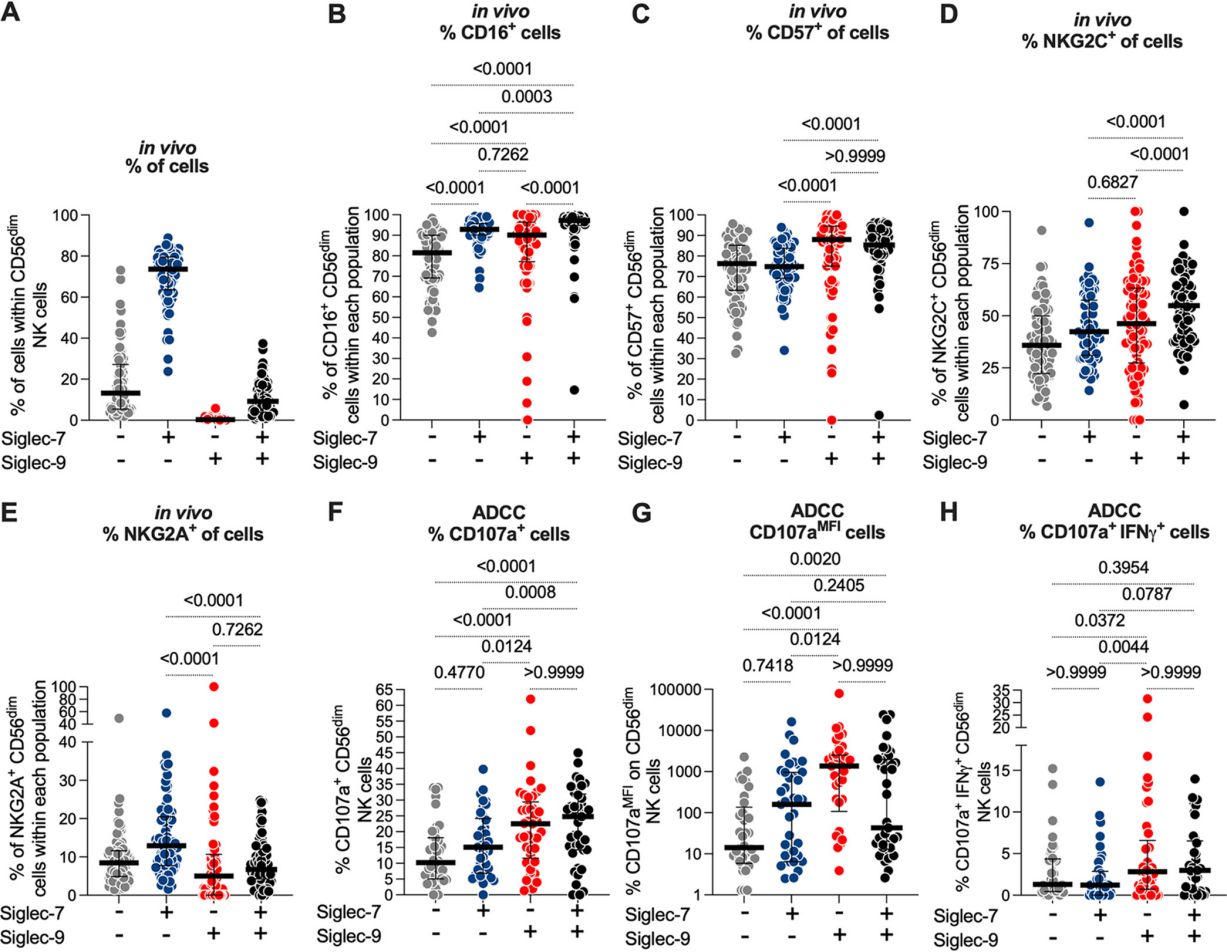
Siglec-9, but not Siglec-7, marks CD56^dim^ NK cells with high ADCC activity against SARS-CoV-2. (A) Percentage of each Siglec-expressing cell subpopulation (Siglec-7^–^ Siglec-9^–^, Siglec-7^+^ Siglec-9^–^, Siglec-7^–^ Siglec-9^+^, or Siglec-7^+^ Siglec-9^+^) within the CD56^dim^ NK cells from all donors (*n* = 79). (B to E) *In vivo* expression of (B) CD16, (C) CD57, (D) NKG2C, and (E) NKG2A on the indicated cell subpopulations (*n* = 79). Median and IQR are shown. Friedman tests with Dunn’s multiple-comparisons correction were used for statistical analyses. (F to H) The ADCC-mediated degranulation of the indicated NK cell subpopulations (*n* = 41) as assessed by (F) the percentage of cells expressing CD107a, (G) the MFI of CD107a, or (H) the percentage of cells expressing CD107a and IFN-γ. Median and IQR are displayed. Friedman tests with Dunn’s multiple-comparisons correction were used for statistical analyses.

10.1128/mbio.03393-22.5 FIG S5 A gating strategy for experiments in Fig. 4 and Fig. 5A to E. Download FIG S5, PDF file, 0.4 MB. Copyright © 2023 Saini et al. 2023 Saini et al. https://creativecommons.org/licenses/by/4.0/ This content is distributed under the terms of the Creative Commons Attribution 4.0 International license.

10.1128/mbio.03393-22.6 FIG S6 Siglec-9 antibody marks Siglec-9^+^ populations with high expression of Siglec-9 mRNA transcripts. To validate the specificity of the Siglec-9 Ab (K8 clone) in identifying Siglec-9^+^ cells, primary NK cells from three healthy individuals were sorted based on Siglec-9 expression into no, low, and high Siglec-9 expression groups. Quantitative PCR (qPCR) was then used to measure the relative copy number of Siglec-9 transcripts in the sorted populations. (A) Gating strategy for the sorting experiments. (B) The relative copy number of Siglec-9 transcripts was measured by qPCR and normalized using the eukaryotic 18S rRNA endogenous control. Relative copy numbers were determined using the comparative threshold cycle (*C_T_*) method. Means and standard error of the mean (SEM) are displayed. Paired *t* tests were used for statistical analysis. Download FIG S6, PDF file, 0.7 MB. Copyright © 2023 Saini et al. 2023 Saini et al. https://creativecommons.org/licenses/by/4.0/ This content is distributed under the terms of the Creative Commons Attribution 4.0 International license.

10.1128/mbio.03393-22.7 FIG S7 Sex-dependent expression of Siglec-7 and Siglec-9 on CD56^dim^ NK cells. To examine the potential impact of sex on Siglec-9 and Siglec-7 expression on CD56^dim^ NK cells, 79 individuals with three COVID-19 disease states (negative; *n* = 12 [grey dots], mild; *n* = 26 [orange dots], and hospitalized; *n* = 41 [maroon dots]), were divided based on sex (female, *n* = 32; male, *n* = 47). Percentages of (A) Siglec-9^+^, (B) Siglec-7^+^, (C) Siglec-9^–^ Siglec-7^–^, (D) Siglec-9^+^ Siglec-7^–^, (E) Siglec-9^–^ Siglec-7^+^, and (F) Siglec-9^+^ Siglec-7^+^ CD56^dim^ NK cells. Median and IQR are shown. Mann-Whitney U tests were used to compare the groups. Download FIG S7, PDF file, 0.6 MB. Copyright © 2023 Saini et al. 2023 Saini et al. https://creativecommons.org/licenses/by/4.0/ This content is distributed under the terms of the Creative Commons Attribution 4.0 International license.

Consistent with previous reports (27), we found that CD56^dim^ NK cells from hospitalized COVID-19 patients, compared to controls, show a reduced percentage of cells expressing CD16 (Fig. S8A), an increased percentage of cells expressing CD57 (Fig. S8B), and a reduced percentage of cells expressing Siglec-7 (Fig. S8C). Focusing on the expression of these markers on Siglec-9^+^, Siglec-9^–^, Siglec-7^+^, and Siglec-7^–^ cells, we found that the Siglec-9^+^ CD56^dim^ NK cells had an activated and mature phenotype, compared to Siglec-9^–^ CD56^dim^ NK cells, as the Siglec-9^+^ cells had higher expression of activation markers/receptors CD16, CD57, and NKG2C and lower expression of the inhibitory receptor NKG2A than the Siglec-9^–^ cells (Fig. 4A). The maturation/activation status of the Siglec-7^+^ CD56^dim^ cells, compared to the Siglec-7^–^ CD56^dim^ NK cells, was less clear. Siglec-7^+^ CD56^dim^ cells had higher levels of CD16 and NKG2C than Siglec-7^–^ CD56^dim^ cells, but no difference in CD57 expression and higher levels of inhibitor marker NKG2A (Fig. 4B). These data suggest that Siglec-9^+^ CD56^dim^ cells are an activated and mature NK subpopulation, which may explain their higher ADCC activity against SARS-CoV-2 Spike-expressing target cells.

10.1128/mbio.03393-22.8 FIG S8 Hospitalized COVID-19 is associated with a decrease in the expression of CD16 and Siglec-7 and an increase in CD57 on CD56^dim^ NK cells. The expression of (A) CD16, (B) CD57, and (C) Siglec-7 on CD56^dim^ NK cells from *n* = 79 (*n* = 12 SARS-CoV-2-negative; *n* = 26 mild COVID-19, and *n* = 41 hospitalized COVID-19). Median and IQR are shown. Kruskal-Wallis tests with Dunn’s multiple-comparisons correction were used for statistical analyses. Download FIG S8, PDF file, 0.2 MB. Copyright © 2023 Saini et al. 2023 Saini et al. https://creativecommons.org/licenses/by/4.0/ This content is distributed under the terms of the Creative Commons Attribution 4.0 International license.

### Siglec-9, but not Siglec-7, marks CD56^dim^ NK cells with high ADCC activity against SARS-CoV-2.

Our data shown in Fig. 2 and 3 suggest that both the Siglec-9^+^ and Siglec-7^+^ CD56^dim^ NK cells exhibit high ADCC activity against SARS-CoV-2. However, Siglec-9 and Siglec-7 are not mutually exclusively expressed on NK cells, and thus there is a population of NK cells which express both Siglec-9 and Siglec-7 (Fig. 5A). Therefore, we next investigated whether NK cells expressing Siglec-9 and/or Siglec-7 have higher ADCC activity against SARS-CoV-2 than do NK cells expressing only Siglec-9 or -7. First, we reanalyzed the *in vivo* phenotype data presented in Fig. 4 to examine the expression of CD16, CD57, NKG2C, and NKG2A on each of the four possible CD56^dim^ NK subpopulations: Siglec-9^–^ Siglec-7^–^, Siglec-9^–^ Siglec-7^+^, Siglec-9^+^ Siglec-7^–^, and Siglec-9^+^ Siglec-7^+^. The data shown in Fig. 5B to E show that the Siglec-9^+^ cells, regardless of Siglec-7 expression, express high levels of CD16, CD57, and NKG2C and low levels of NKG2A. On the other hand, the Siglec-7^+^ cells, regardless of Siglec-9 expression, express high levels of CD16 but not CD57 or NKG2C. Furthermore, the Siglec-7^+^ cells express high levels of the inhibitory receptor NKG2A. These data further suggest that Siglec-9, but not Siglec-7, marks cells with activated and mature phenotypes.

We next examined the ADCC potential of each of the four NK subpopulations by reanalyzing the data presented in Fig. 2 and 3. The data shown in Fig. 5F to H suggest that CD56^dim^ NK cells which express Siglec-9, regardless of their Siglec-7 expression, have higher ADCC ability against SARS-CoV-2 than cells that do not express Siglec-9. Together, these data suggest that NK cells expressing Siglec-9 exhibit an activated and mature phenotype, which may contribute to their high ADCC toward SARS-CoV-2 Spike-expressing target cells.

### Blocking Siglec-9 interactions, using a Siglec-9-blocking antibody, enhances the anti-SARS-CoV-2 ADCC of CD56^dim^ NK cells.

**FIG 6:**
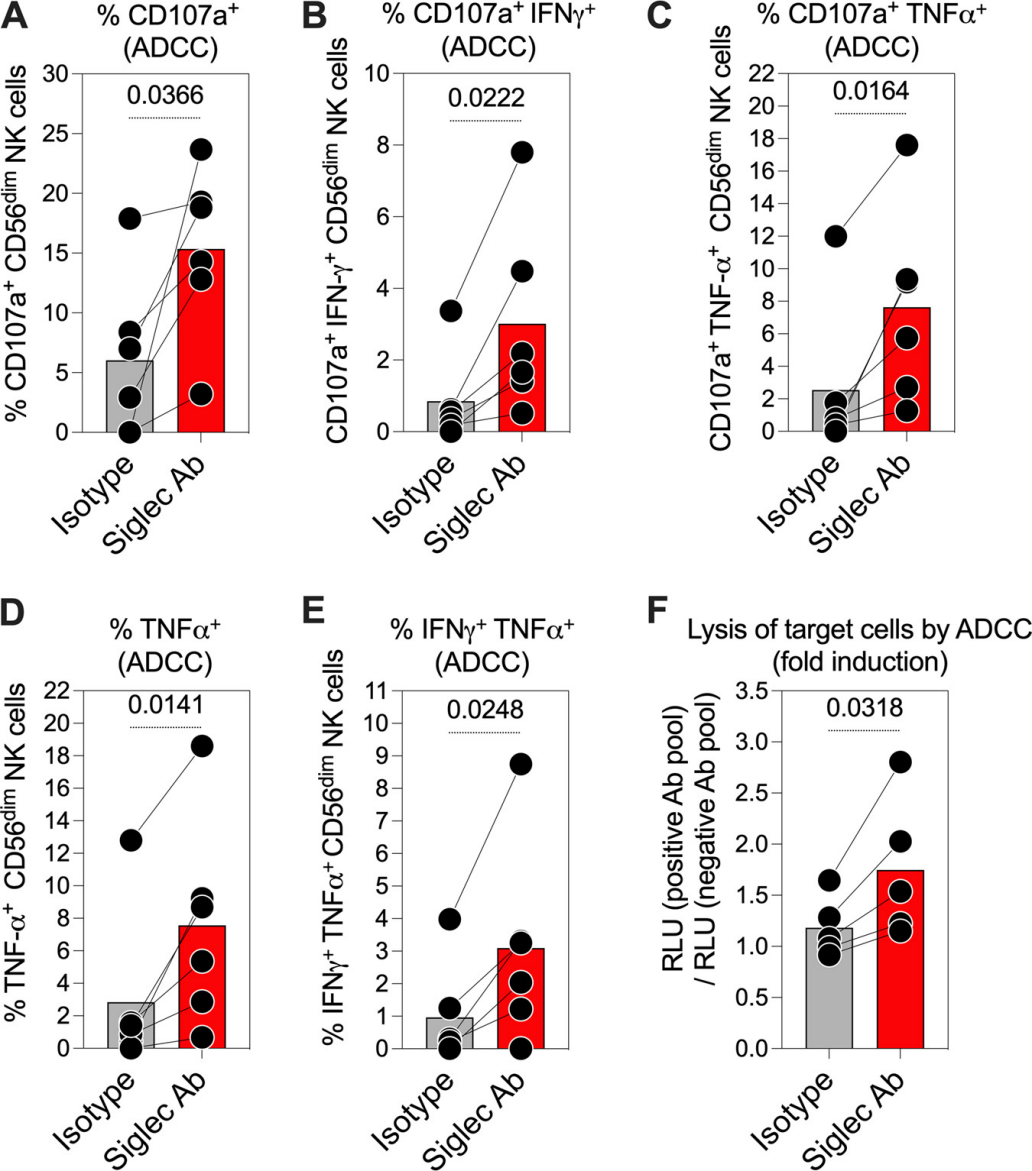
Blocking Siglec-9 interactions, using a Siglec-9-blocking antibody, enhances the anti-SARS-CoV-2 ADCC of CD56^dim^ NK cells. (A to E) The impact of the Siglec-9-blocking antibody, compared to an isotype control, on ADCC-mediated NK degranulation and/or cytokine production against SARS-CoV-2. PBMCs from six healthy controls were used as effector cells, and SARS-CoV-2 Spike-expressing 293T cells were used as target cells. Cells were cocultured at a 10:1 (E:T) ratio for 12 h. NK degranulation and/or cytokine production was assessed as the percentage of cells expressing (A) CD107a, (B) CD107a and IFN-γ, (C) CD107a and TNF-α, (D) TNF-α, (E) and IFN-γ and TNF-α. Paired *t* tests were used for statistical analysis. (F) The impact of the Siglec-9-blocking antibody, compared to an isotype control, on the ADCC-mediated lysis of SARS-CoV-2 target cells. Purified NK cells isolated from the PBMCs of five healthy donors were used as effector cells, and SARS-CoV-2 S CHO-K1 cells were used as target cells. Cells were cocultured at a 5:1 E:T ratio for 5 h. The SARS-CoV-2 S CHO-K1 cells stably express the SARS-CoV-2 Spike (S) protein and a HaloTag-HiBiT protein. When the target cells are lysed by ADCC, the intracellular HaloTag-HiBiT protein interacts with the extracellular detection reagent to generate a luminescence signal that can quantitatively measure the degree of target cell lysis. A paired *t* test was used for statistical analysis.

Our data thus far suggest that NK cells expressing Siglec-9 exhibit high ADCC activity; however, the Siglec-9 molecule itself is an established inhibitory receptor which functions as a glyco-immune checkpoint to restrict NK cytotoxicity (13, 16, 18). Therefore, we next asked whether blocking the inhibitory signaling of Siglec-9, using an in-house Siglec-9-blocking antibody, would further enhance the ADCC activity of Siglec-9^+^ NK cells against SARS-CoV-2. First, we examined the impact of the Siglec-9-blocking antibody, compared to an isotype control, on the ADCC-mediated NK degranulation against SARS-CoV-2 (Fig. 6A to E). For these experiments, we used PBMCs from six healthy controls as effector cells and 293T cells expressing SARS-CoV-2 Spike as target cells. Blocking Siglec-9 significantly enhanced the ADCC potential of CD56^dim^ NK cells as shown by the increased percentages of CD107a^+^ (Fig. 6A), CD107a^+^ IFN-γ^+^ (Fig. 6B), CD107a^+^ TNF-α^+^ (Fig. 6C), TNF-α^+^ (Fig. 6D), or IFN-γ^+^ TNF-α^+^ (Fig. 6E) CD56^dim^ NK cells.

Next, we examined whether the enhanced NK degranulation/cytokine production caused by blocking Siglec-9 resulted in increased lysis of target cells. For these experiments, we used purified NK cells isolated from the PBMCs of five healthy donors as effector cells and SARS-CoV-2 S CHO-K1 cells as target cells. As shown in Fig. 6F, blocking Siglec-9 enhanced the ADCC-mediated lysis of target cells compared to the isotype control. These data show that the Siglec-9 interactions indeed restrain the ADCC activity of the already highly activated and cytotoxic Siglec-9^+^ NK cells and suggest that strategies to target Siglec-9 may enhance NK cytotoxicity against SARS-CoV-2.

## DISCUSSION

Previous reports have associated severe COVID-19 with alterations to NK cell profiles (27). However, whether these alterations result in a dysfunctional anti-SARS-CoV-2 NK cytotoxicity has not been fully studied. In this report, we first performed functional assays to examine both the direct cytolytic and ADCC-mediated degranulation of NK cells from hospitalized COVID-19 patients and controls. Our data suggest that severe COVID-19 is not only associated with alterations to the phenotypic profiles of NK cells, but also with reductions in their cytolytic and ADCC activity. The mechanisms underlying these dysfunctions are unclear; however, severe COVID-19 is associated with a state of hyperinflammation, characterized by an ensuing cytokine storm (1, 28) and dysregulated myeloid cell functions (29, 30). NK cell functions can be significantly modulated by the cytokine milieu (31–34) and interactions with myeloid cells (30). Whether deregulated secretion of cytokines, such as TGF-β (transforming growth factor β) (35), and myeloid cell dysfunction directly and/or indirectly contribute to the diminished NK functions during severe COVID-19 warrants further investigation. The causative versus consequential effects of diminished NK functions and COVID-19 severity are also unknown. Studies in animal models of SARS-CoV-2 infection will be needed to explore this potential link. However, knowing that the anti-SARS-CoV-2 cytolytic activity of NK cells is likely compromised during severe COVID-19, regardless of the contribution of these functions to disease severity, suggests a need to develop strategies to enhance NK function during severe infections with SARS-CoV-2, and other similar emerging viruses, to control infection and decrease disease severity. These strategies could be particularly important for immunocompromised individuals who may need an immunotherapeutic approach to help control viral infections (36–42).

Identifying NK subpopulations capable of targeting virus-infected cells could be an essential step in developing efficient strategies to enhance NK cytotoxicity against SARS-CoV-2 and other viral infections. In this report, we focused on NK cells expressing Siglec-7 and/or Siglec-9. Siglecs are emerging ITIM-containing, major histocompatibility complex (MHC)-independent inhibitory receptors that control host immune responses by interacting with sialoglycans on the surface of target cells. Siglec-7 is expressed on almost all NK cells and binds to α2-8 Sialic acid, whereas Siglec-9 is selectively expressed on a subset of CD56^dim^ NK cells and binds to α2-3 Sialic acid (13, 43). We identified the Siglec-7^–^ CD56^dim^ NK subpopulation, which is accumulated during severe COVID-19, as a dysfunctional NK subpopulation during SARS-CoV-2 infection. This is consistent with previous reports describing decreased levels of Siglec-7 as a marker for dysfunctional NK cells during HIV infection (24–26). In addition to Siglec-7, we also identified the Siglec-9^+^ CD56^dim^ NK subpopulation, which has never been implicated during SARS-CoV-2 infection, as a highly cytotoxic NK subpopulation. This is also consistent with previous reports that this NK subpopulation exhibits high antiviral activity during HIV infection (13).

The Siglec-9^+^ CD56^dim^ NK cells have an activated phenotype (higher expression of activating receptors and lower expression of inhibitory receptors) during cancer (16), HBV infection (18), and HIV infection (13). Indeed, we found that the Siglec-9^+^ CD56^dim^ NK exhibits an activated phenotype with higher levels of activating/maturation receptors and markers and lower expression of the inhibitory receptor NKG2A, compared to Siglec-9^–^ CD56^dim^ NK cells, during SARS-CoV-2 infection. Based on our results, these cells have an activated phenotype even in healthy controls, suggesting that this cell population is naturally activated with potential cytotoxic capacity and could be exploited against several viral and nonviral infections.

**FIG 7:**
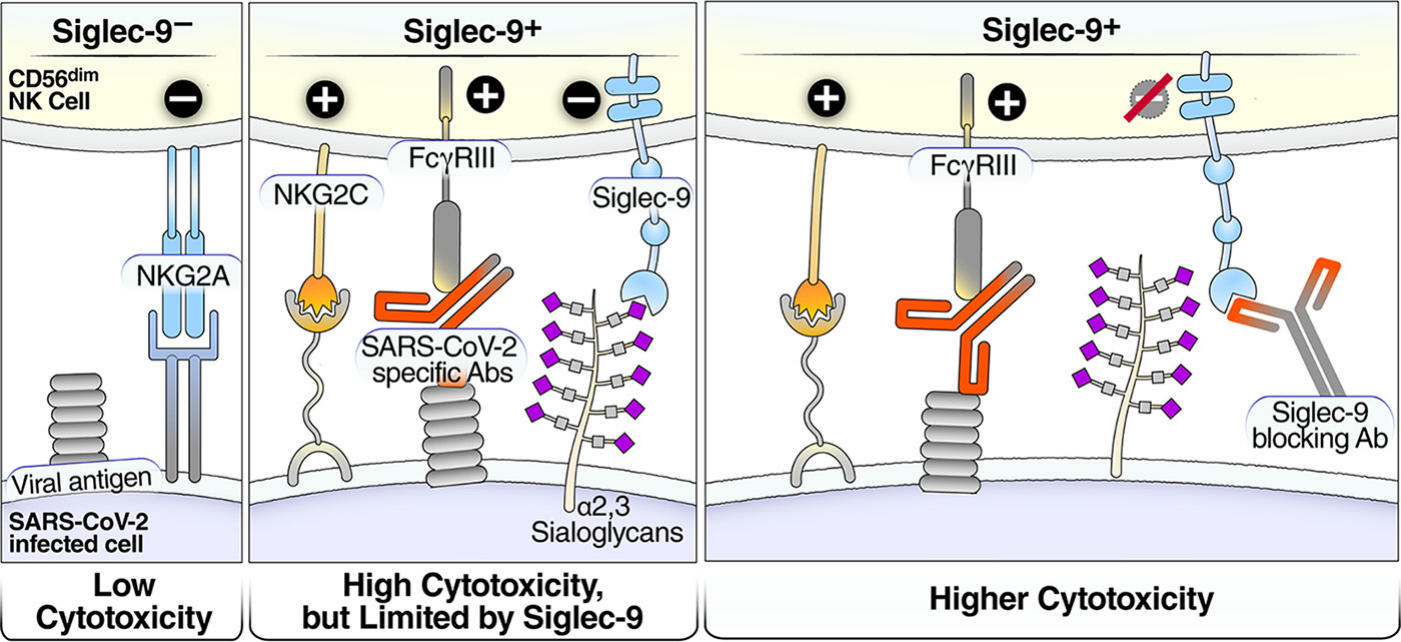
Model of how a Siglec-9-blocking antibody increases the cytotoxicity of Siglec-9^+^ NK cells. Left panel: Siglec-9^–^ cells have low cytotoxicity. Middle panel: the Siglec-9^+^ CD56^dim^ NK subset has high ADCC activity, possibly due to elevated expression of CD16 (FcγRIII; a mediator of ADCC activity), CD57 (maturation marker), and NKG2C (activating receptor), and reduced expression of the inhibitory receptor NKG2A, compared to Siglec-9^–^ CD56^dim^ NK cells. However, the Siglec-9 molecule itself is an inhibitory receptor which restrains the cytolytic ability of these highly cytotoxic Siglec-9^+^ CD56^dim^ NK cells by binding to Sialic acid on the surface of target cells. Right panel: blocking the inhibitory receptor, Siglec-9, using a blocking antibody can unleash a higher ADCC potential of the Siglec-9^+^ CD56^dim^ subpopulation.

The highly activated phenotype of the Siglec-9^+^ CD56^dim^ NK cells is consistent with our functional analysis demonstrating that Siglec-9^+^ NK cells exhibit higher ADCC than Siglec-9^–^ NK cells. These results are consistent with the highly cytotoxic nature of Siglec-9^+^ NK cells. However, the Siglec-9 receptor itself is an inhibitory receptor that restrains the cytolytic ability of these otherwise highly cytotoxic Siglec-9^+^ NK cells. The binding of Siglec-9 to α2-3 Sialic acid on target cells induces an inhibitory signal transduction cascade by recruiting the tyrosine phosphatase SHP-1, which counteracts the phosphorylation-mediated activation of other signaling molecules (44, 45). Indeed, blocking Siglec-9 further enhanced the ability of NK cells to kill target cells expressing SARS-CoV-2 antigen by ADCC. This result is consistent with the known inhibitory function of the Siglec-9 molecule itself on these otherwise cytotoxic cells. Our data support a model in which Siglec-9^+^ CD56^dim^ NK cells are cytotoxic but are restrained by the inhibitory nature of Siglec-9 receptor signaling (Fig. 7, left two panels). Furthermore, our data suggest that blocking Siglec-9 interactions is a promising strategy to unleash the full potential of the Sigec-9^+^ NK subpopulation that is otherwise highly cytotoxic (Fig. 7, right panel).

In this study, we highlighted the Siglec-9/sialic acid axis as a glyco-immune checkpoint mechanism that viral infections may exploit to evade immune surveillance by cytotoxic Siglec-9^+^ NK cells. We examined the potential of Siglec-9-blocking antibody to enhance the anti-SARS-CoV-2 ADCC activity. Indeed, in proof-of-concept experiments, we found that blocking Siglec-9, using a blocking antibody, enhanced the anti-SARS-CoV-2 specific ADCC activity of NK cells *in vitro*. In the last few years, several Siglec-blocking antibodies have been tested for their ability to prevent Siglec-mediated inhibition of immune functions. Blocking antibodies against Siglec-7 and Siglec-9 enhances anti-tumor immune activity both *in vitro* and *in vivo* (15, 16, 46–49). Similarly, blocking Siglec-9 interactions enhanced NK cytotoxicity against HIV-infected targets (13) and reversed the dysfunctionality of NK cells during HBV infection (18). These blocking antibodies are promising tools to enhance NK cytotoxicity against virally infected cells. However, mono-specific antibodies blocking Siglecs may possibly induce nonspecific inflammation because Siglecs are expressed on other immune cells, including myeloid cells (20, 49, 50), and play an important role as immune checkpoints against hyperinflammation and autoimmunity (50, 51). For instance, it was recently shown that Siglec-9 interactions on neutrophils play an important role in modulating inflammation during COVID-19 (52). Therefore, likely bi-specific antibodies which target the blocking antibody to specific immune cells (NK cells) and/or virally infected cells will be needed to utilize the potential positive effects of the Siglec blockade on enhancing anti-SARS-CoV-2 NK immune functions while avoiding any potential nonspecific inflammatory side effects.

This study has limitations. First, we focused our analyses on NK cells from the peripheral blood; in future studies, it will be important to examine the frequency and phenotype of Siglec-7^+^ CD56^dim^ cells and Siglec-9^+^ CD56^dim^ cells in bronchoalveolar lavage samples and different tissues. Furthermore, given our results demonstrating sex-dependent differences in Siglec expression on NK cells and NK cytotoxic activities, larger studies are needed to examine the impact of sex/gender (and other demographic and clinical features) on the relationship between Siglec expression and antiviral immune functions. In addition, we have used the plasma N-antigen load as a simple blood-based biomarker of SARS-CoV-2 infection severity (21–23); however, further examinations are needed to establish links between NK functionality and upper-respiratory viral load. Second, regarding the NK cytotoxicity assays, it will be critical in future studies to explore the cytotoxicity of NK cells against primary epithelial cells infected with different variants of SARS-CoV-2. Finally, the proof-of-concept experiments shown in Fig. 6, using the Siglec-9 blocking antibody, were exploratory in nature; examining the utility of this blocking antibody *in vivo* using animal models of SARS-CoV-2 infection will be needed. In addition, and as mentioned above, it is likely that developing a bi-specific version of these antibodies to target them to specific immune cells and/or virally infected cells will be needed to utilize their beneficial effects on NK cells while avoiding any potential side effects on other immune cells. Despite these shortcomings, this study is the first to describe Siglec-9^+^ CD56^dim^ NK cells as an NK subpopulation that can be exploited to develop novel immunotherapeutic tools against SARS-CoV-2-infected cells.

## MATERIALS AND METHODS

### Ethics.

Research protocols were approved by the institutional review board (IRB) at Rush University (IRB approval no. 20032309). All experimentation was conducted following the US Department of Health and Human Services guidelines.

### Characteristics of the study cohort.

We used PBMCs and plasma from 67 individuals who tested positive for SARS-CoV-2 (by PCR) and 12 negative controls. The 67 SARS-CoV-2-positive individuals were either outpatients (mild; *n* = 26) or inpatients (hospitalized; *n* = 41) (Table S1). Samples from hospitalized patients were collected when patients were admitted to the hospital.

### Direct cytotoxicity assay against SARS-CoV-2 Spike-expressing 293T cells.

Frozen PBMCs were thawed in complete growth medium (RPMI with 10% fetal bovine serum [FBS]) and rested overnight. PBMCs (1 × 10^6^) were then cocultured with Spike-expressing 293T (S-293T) target cells (1 × 10^5^) at a 10:1 (E:T) ratio in a complete growth medium in the presence of GolgiStop and anti-CD107a PE antibody (BD Biosciences). The cocultured cell mixture was then pelleted at 200 × *g* for 2 min and incubated at 37°C for 16 h. Upon incubation, cells were stained for the following surface markers: CD56 (APC-Cy7; BioLegend), CD3 (Alexa-488; BD Biosciences), Siglec-9 (APC; BioLegend), or Siglec-7 (Alexa-700; BioLegend). Cells were washed twice, fixed (using Cytofix/Cytoperm; BD Biosciences), and permeabilized (Perm/Wash buffer; BD Biosciences). Following permeabilization, cells were intracellularly stained for IFN-γ (BV421; BD Biosciences) and TNF-α (PE-Dazzle 594; BioLegend). At least 100,000 events were acquired by flow cytometry on a BD Biosciences LSR II Flow Cytometer. Cytolytic NK cells were gated as CD3^−^ and CD56^dim^ (Fig. S1A). Direct cytotoxicity was calculated by subtracting the background NK degranulation/cytokine production of the PBMC-only culture from the NK degranulation/cytokine production of the PBMC and target cell cocultures (Fig. 1A, left).

### ADCC assay against SARS-CoV-2 Spike-expressing 293T cells.

IgG was isolated from the plasma of the donors using the Pierce Protein G Spin Plate for IgG (Thermo Fisher Scientific) kit. Purified IgG was quantified using NanoDrop (absorbance of *A*_280_). Purified IgGs from the SARS-CoV-2-negative donors were pooled in equal concentrations to obtain a negative pool. Purified IgGs from SARS-CoV-2-positive donors were pooled in equal concentrations to obtain a positive pool. The ADCC assay was performed identically to the direct cytotoxicity assay but with the target cells pre-incubated (for 15 min) with the negative or positive pools (at 10 μg/well) before being cocultured with the PBMCs from each donor. ADCC was then assessed by subtracting the NK degranulation of the coculture with the negative antibody pool from NK degranulation of the coculture with the positive antibody pool (after subtracting background NK degranulation) (Fig. 1A, right).

### Target cell lysis of Spike-expressing CHO-K1 using PBMCs.

Target cell lysis was performed using the Promega HaloTag-HiBit ADCC kit, following the manufacturer's instructions. Briefly, cryopreserved PBMCs and spike-expressing CHO-K1 cells were thawed, rested overnight. Upon resting, CHO-K1 target cells were incubated with either the SARS-CoV-2-positive or SARS-CoV-2-negative IgG pools at 0.5 μg/well concentration. After 15 min, PBMC cells (2.5 × 10^4^) and CHO-K1 (2,500) target cells were cocultured at a 10:1 (E:T) ratio, in complete growth medium for 5 h. After 5 h, the substrate was added, and luminescence was measured after an additional 10 min. Luminescence values of each donor for the positive SARS-CoV-2 IgG pool were subtracted from those obtained from the respective negative SARS-CoV-2 IgG pool to obtain the specific target cell lysis.

### Direct cytotoxicity assay against K562 cells.

Frozen PBMCs were thawed in complete growth medium (RPMI with 10% FBS) and rested overnight. PBMCs (1 × 10^6^) were then cocultured with K562 target cells (2 × 10^5^) at 5:1 (E:T) ratio in a complete growth medium in the presence of GolgiStop (BD Biosciences) and anti-CD107a PE antibody (BD Biosciences). The cocultured cell mixture was then pelleted at 200 × *g* for 2 min and incubated at 37°C for 3 h. Upon incubation, cells were stained with the same antibodies as described above, fixed, permeabilized, and intracellularly stained for IFN-γ and TNF-α. At least 100,000 events were acquired by flow cytometry on a BD Biosciences LSR II Flow Cytometer. Cytotoxicity was calculated by subtracting the background NK degranulation/cytokine production of the PBMCs alone culture from the NK degranulation/cytokine production of the cocultures of the PBMCs and target cells.

### SARS-CoV-2 N-antigen quantification.

The SARS-CoV-2 N-antigen plasma load was quantified using a Single Molecular Array (Simoa) immunoassay on the Simoa HD-X analyzer (Quanterix), as previously described (23).

### Phenotypic characterization of Siglec-7^+^ and Siglec-9^+^ CD56^dim^ NK cells.

Phenotypic characterization of NK cells expressing Siglec-7 and Siglec-9 was performed on cryopreserved PBMC (*n* = 79) from the study cohort by multiparameter flow cytometry. In brief, cryopreserved PBMC were thawed in prewarmed RPMI (RPMI 1640 medium; Mediatech) supplemented with 10% heat-inactivated FBS (Sigma), 1% penicillin-streptomycin (Lonza), and 2 mM l-glutamine (Sigma) and collected by centrifugation. Cells were then washed in Dulbecco’s phosphate-buffered saline (DPBS) without Ca^++^\Mg^++^ (DPBS-CMF) and collected by centrifugation. Next, the cells were stained with an Aqua Live/Dead Cell Stain kit (Invitrogen) to assess their viability, washed in DPBS-CMF, and held for cell-surface staining. Cells were then incubated with a cocktail of fluorochrome-conjugated anti-human monoclonal antibodies: CD3 AF700, CD19 AF700, CD14 AF700, HLA DR APC-H7, CD56 PE-Cy7, CD16 BV605, CD57 FITC, CD38 PE-CF594, NKG2A BB700, NKG2C BV786, and CD161 (BV421) from BD Biosciences along with Siglec-9 APC and Siglec-7 PE from Biolegend. Cells were washed in fluorescence-activated cell sorter (FACS) buffer (DPBS-CMF + 0.5% bovine serum albumin [BSA] + 0.1% Na Azide) and then fixed in 1% paraformaldehyde (PFA, Polysciences) before acquisition on an LSRFortessa SORP flow cytometer (BD Biosciences). Data were analyzed using FlowJo Software version 9.9.6 (Treestar Inc.). Gating strategy is described in Fig. S5.

### Sorting CD56^dim^ NK cells based on their Siglec expressing and measuring relative copy number of Siglec-9 mRNA using qPCR.

Around ~10 million primary NK cells were negatively selected from PBMCs isolated from three healthy donors using a Human EasySep NK Isolation kit as per the manufacturer’s instructions (StemCell Technologies). Cells were then stained for CD56 (APC-Cy7; Biolegend), CD3 (Alexa-488; BD Biosciences), Siglec-9 (APC; Biolegend). CD3^−^ CD56^dim^ NK cells were sorted into three populations with no, low, and high Siglec-9 expression using the FACSymphony S6 SE (FACSAriaII) (Fig. S6).

Sorted cells were lysed, and total RNA was extracted from them using the RNAeasy Minikit with on-column DNase treatment (Qiagen) according to the manufacturer’s instructions. cDNA was generated using SuperScript VILO MasterMix (Invitrogen) according to the manufacturer’s instructions. The relative copy number of Siglec-9 transcripts was quantified in a qPCR containing 4 pmol of each Siglec-9-specific primer and probe (Life Technologies, assay ID no. Hs00534924_m1), 10 μL 2× TaqMan Universal Master Mix (Applied Biosystems), and 5 μL diluted cDNA. Reactions were performed in a QuantStudio 6 Flex Real-Time PCR system (Applied Biosystems) using the following cycling conditions: 50°C for 2 min, 95°C for 10 min followed by 45 cycles of 95°C for 15 s, and 60°C for 1 min. Data were normalized using the eukaryotic 18S rRNA endogenous control (Applied Biosystems) as a housekeeping gene. Relative copy numbers were determined using the comparative threshold cycle (*C_T_*) method (53).

### Generation and characterization of human Siglec-9-blocking antibody.

Transgenic H2L2 mice (Harbor BioMed, Cambridge, MA) which encode the human immunoglobulin repertoire were used for immunization (54). Immunization and antibody sequencing were performed similarly to a detailed protocol described recently using transgenic H2L2 mice (55). Briefly, mice were immunized with 50 μg of DNA encoding human Siglec-9 two times at 2-week intervals. Mice then received two booster injections at 2-week intervals: the first contained Siglec-9 DNA and the second contained 50 μg of purified recombinant human Siglec-9 protein (R&D Systems). Murine SP2/0-Ag14 (SP2/0) myeloma cell lines were used to generate hybridomas by the chemical fusion of splenocytes from immunized mice. After antibody-binding confirmation using enzyme-linked immunosorbent assay (ELISA), mouse splenocytes were used to generate hybridomas and sequence antibodies, as described recently in detail (49, 55).

For recombinant expression in mammalian cells, antibody constructs were cloned into the pCDNA3.4 expression vector. Gene constructs encoding full-length IgG were designed (GenScript), and transient production in suspension HEK293 cells was performed in serum-free suspension culture to express the full-size antibodies. The reactivity and specificity of the recombinant anti-Siglec-9 antibody were examined by ELISA. The ELISA plates were coated with human recombinant Siglec-9 protein (1 μg/mL) (R&D Systems), human recombinant Siglec-7 protein (1 μg/mL; negative control) (R&D Systems), or HIV gp120 protein (1 μg/mL; as a negative control) overnight at 4°C. After being washed with PBS and blocked by 3% BSA, the purified anti-Siglec-9 antibody was added at different dilutions and incubated for 1 h at room temperature. The wells were then washed and detected by 3,3′,5,5′-tetramethylbenzidine substrate after incubation with goat anti-mouse secondary antibody. The reaction was stopped by the addition of 1 M H_2_SO_4_, and absorbance was measured at 450 nm by an ELISA reader (Fig. S9).

10.1128/mbio.03393-22.9 FIG S9 Siglec-9 blocking antibody characterization. The binding of different dilutions of the recombinantly expressed anti-Siglec-9 antibody to: (i) recombinant Siglec-9 protein, (ii) recombinant Siglec-7 protein (as a negative control), and (iii) HIV-1-gp120 protein (as a negative control) was determined by enzyme-linked immunosorbent assay (ELISA). Each point represents the Optical density (OD) value (mean ± SEM). Download FIG S9, PDF file, 0.1 MB. Copyright © 2023 Saini et al. 2023 Saini et al. https://creativecommons.org/licenses/by/4.0/ This content is distributed under the terms of the Creative Commons Attribution 4.0 International license.

### ADCC-mediated NK degranulation assay in the presence of Siglec-9-blocking antibody.

Cryopreserved PBMCs from six healthy donors were thawed, rested overnight, and incubated with an in-house Siglec-9-blocking antibody at 0.5 μg/well for 15 min. S-293T target cells (1 × 10^5^) were incubated with either the positive or negative pool at 0.5 μg/well concentration. After 15 min, pretreated PBMCs (1 × 10^6^) and S-293T cells were cocultured at 10:1 effector-to-target ratio, and degranulation was examined as previously described.

### Human NK cell isolation and target cell lysis in the presence of Siglec-9 blocking antibody.

NK cells were isolated by negative selection from PBMCs obtained from five healthy donors using the EasySep Human NK Cell isolation kit (STEMCELL Technologies) following the manufacturer’s protocol. Target cell lysis was performed using the Promega HaloTag-HiBit ADCC kit, following the manufacturer’s instructions. Briefly, isolated NK cells were incubated with the Siglec-9 antibody (at 0.5 μg/well) for 15 min. CHO-K1 cells were also pre-incubated with either the positive or negative pool at 0.5 μg/well for 15 min. CHO-K1 (2,500) and NK cells (1.25 × 10^4^) were then cocultured at a 5:1 effector-to-target ratio in complete growth medium for 5 h. After 5 h, the substrate was added, and luminescence was measured after 10 min. Luminescence values of each donor for the positive pool were divided by the values obtained from the respective negative pool to obtain the specific target cell lysis.

### Statistical analysis.

Kruskal-Wallis tests with Dunn’s multiple-comparisons correction were used for statistical analyses in Fig. 1B to I, Fig. S1B to G, Fig. S3B to F, and Fig. S8. Spearman’s rank-order correlations were used for the statistical analyses in Fig. 1J to M, Fig. 2J, and Fig. 3J. Wilcoxon’s signed-rank tests were used for the statistical analyses in Fig. 2A to I, Fig 3A to I, Fig. 4 (to compare cells within each disease group), and Fig. S4. Mann-Whitney U tests were used to compare cells between the different group states shown in Fig. 4 and were also used for the statistical analyses in Fig. S2, Fig. S3A, and Fig. S7. Friedman tests with Dunn’s multiple-comparisons correction were used for the statistical analyses in Fig. 5B to H. Paired *t* tests were used for the analyses shown in Fig. 6 and Fig. S6. Data were analyzed using Prism 9.0 (GraphPad Software).
