# Supplementary figures and images for: Siglec-9 Restrains Antibody-Dependent Natural Killer Cell Cytotoxicity against SARS-CoV-2
Source: mBio. 2023 Feb 2;14(1):e03393-22. doi: 10.1128/mbio.03393-22 (PMC9973332; doi:10.1128/mbio.03393-22)

## Supplementary Figure 1

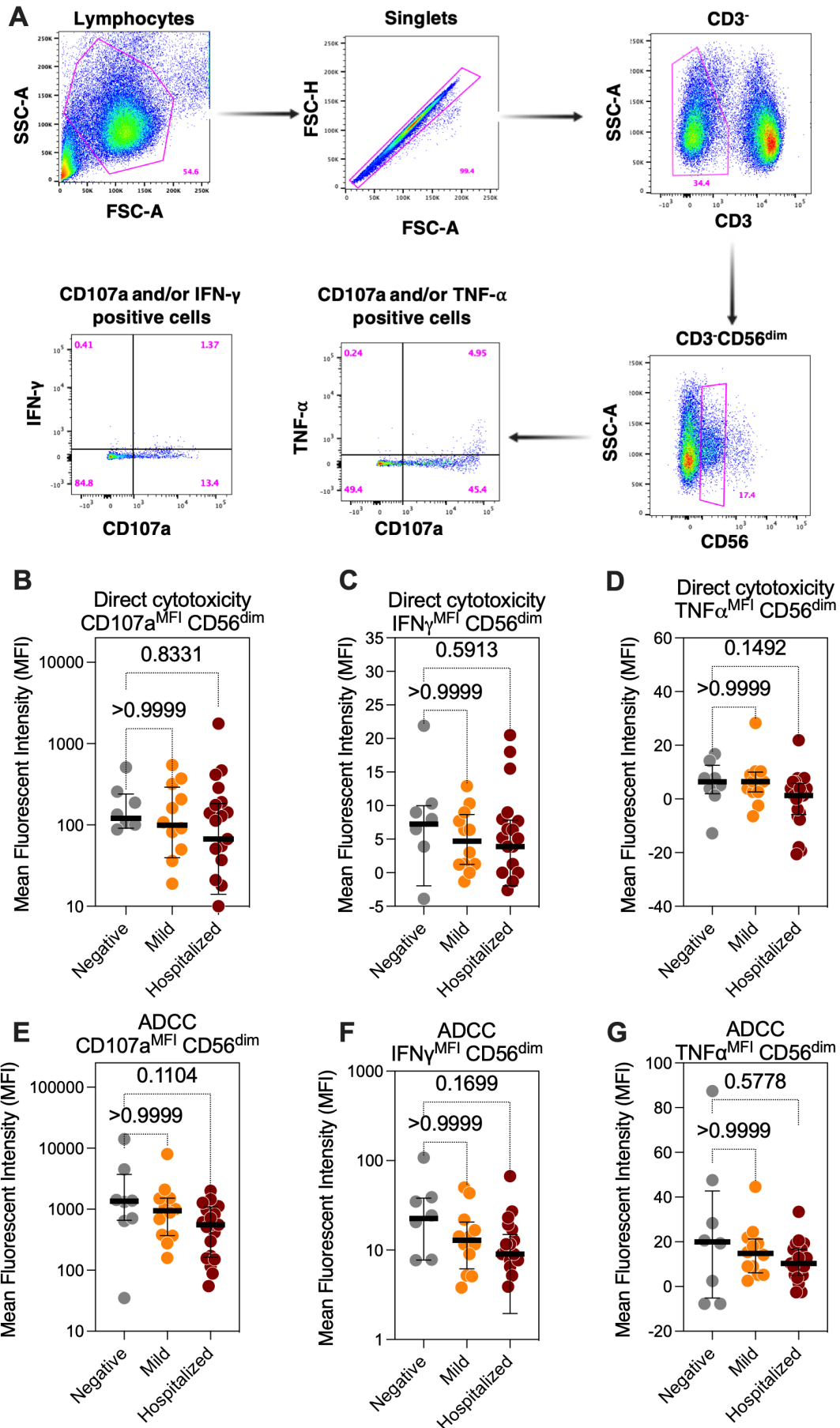

Supplement: FIG S1 [file mbio.03393-22-s0001.pdf]

Supplementary Figure 2

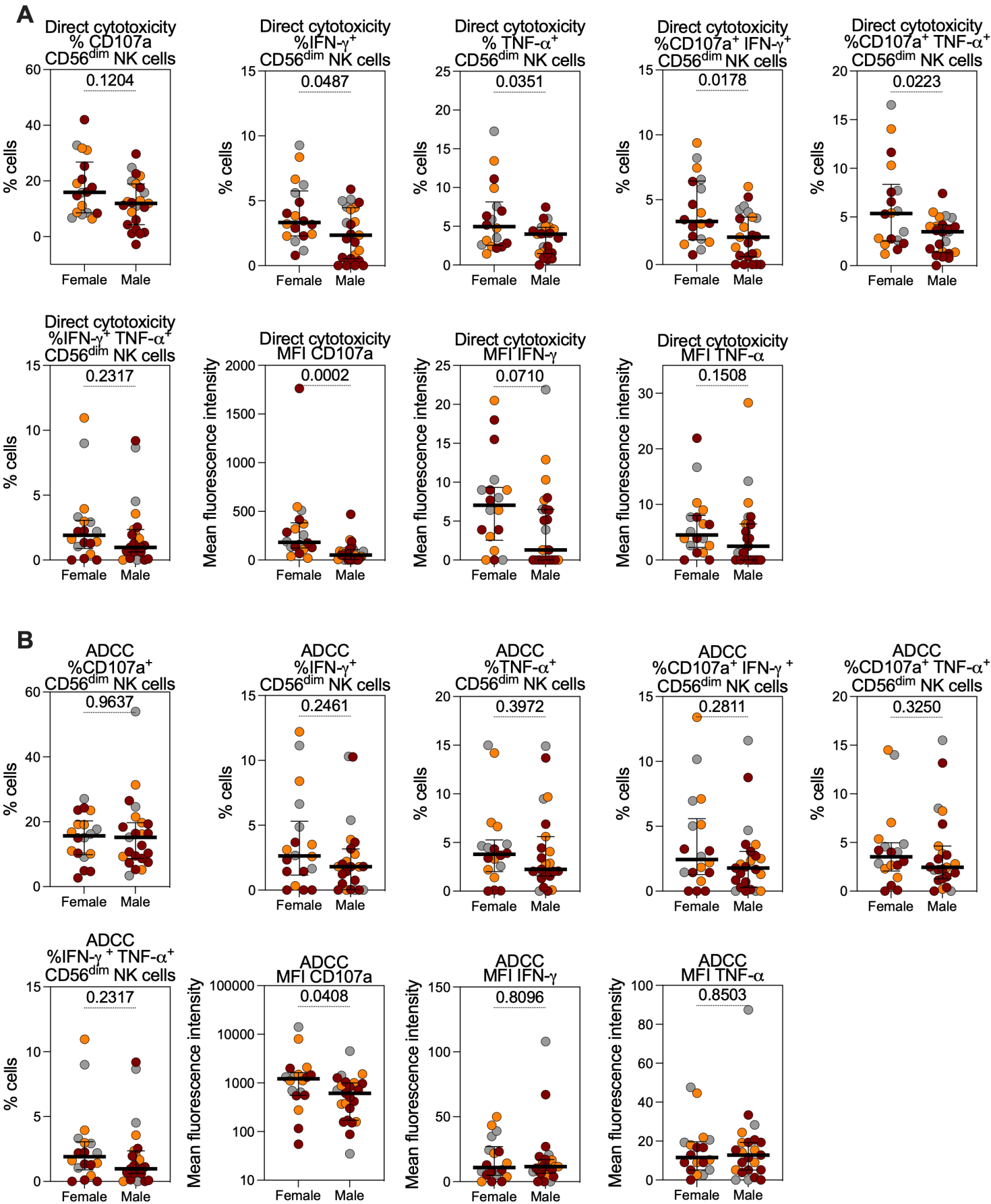

Supplement: FIG S2 [file mbio.03393-22-s0002.pdf]

# Supplementary Figure 3

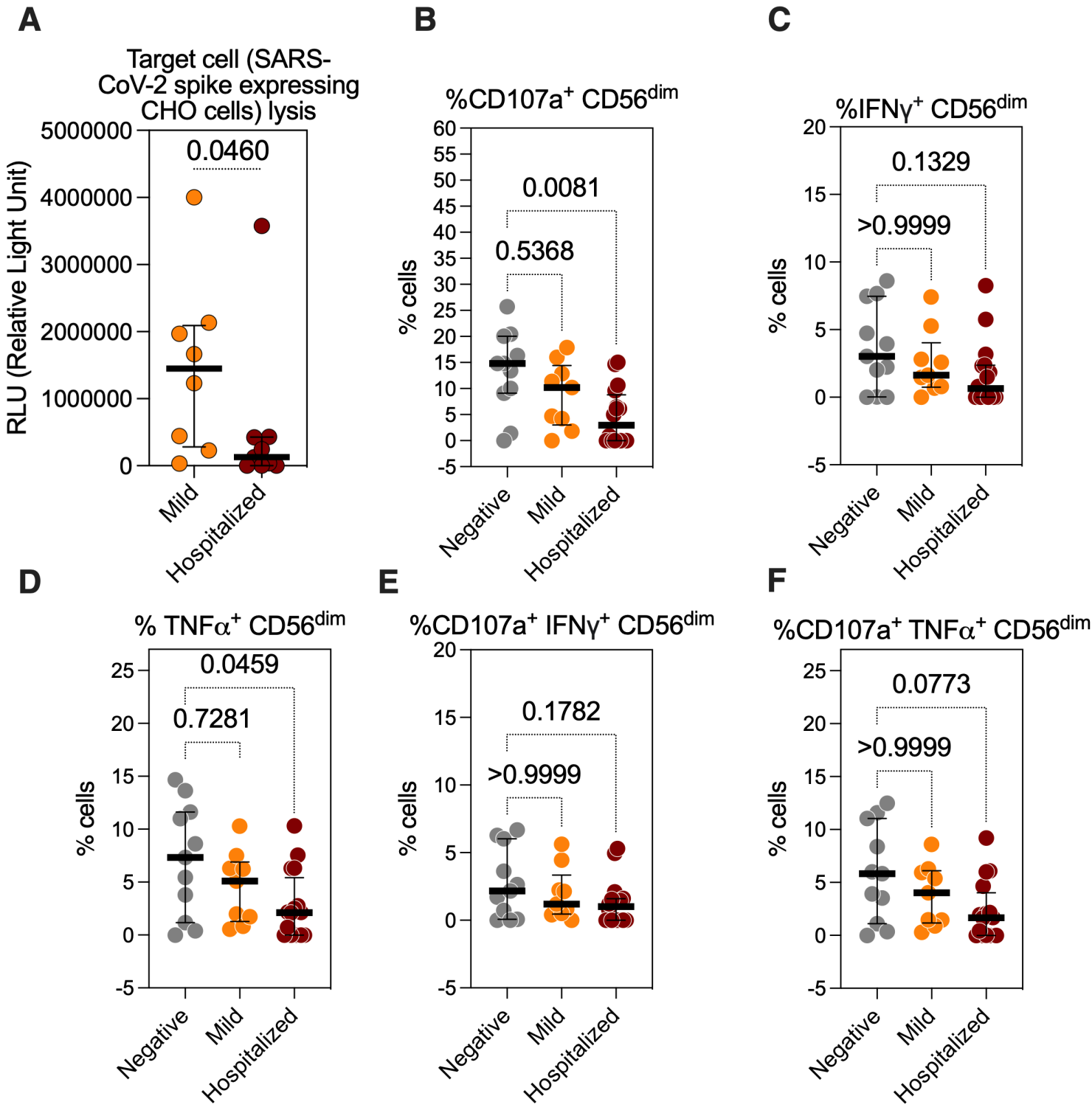

Supplement: FIG S3 [file mbio.03393-22-s0003.pdf]

Supplementary Figure 4

● Siglec-7<sup>+</sup> CD56<sup>dim</sup> NK cells      ○ Siglec-7<sup>-</sup> CD56<sup>dim</sup> NK cells

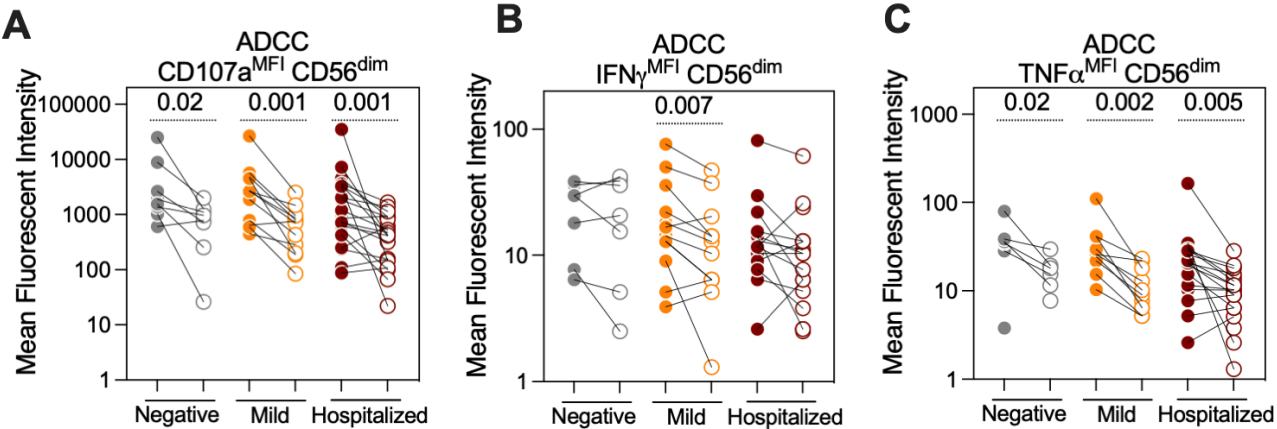

Supplement: FIG S4 [file mbio.03393-22-s0004.pdf]

Supplementary Figure 5

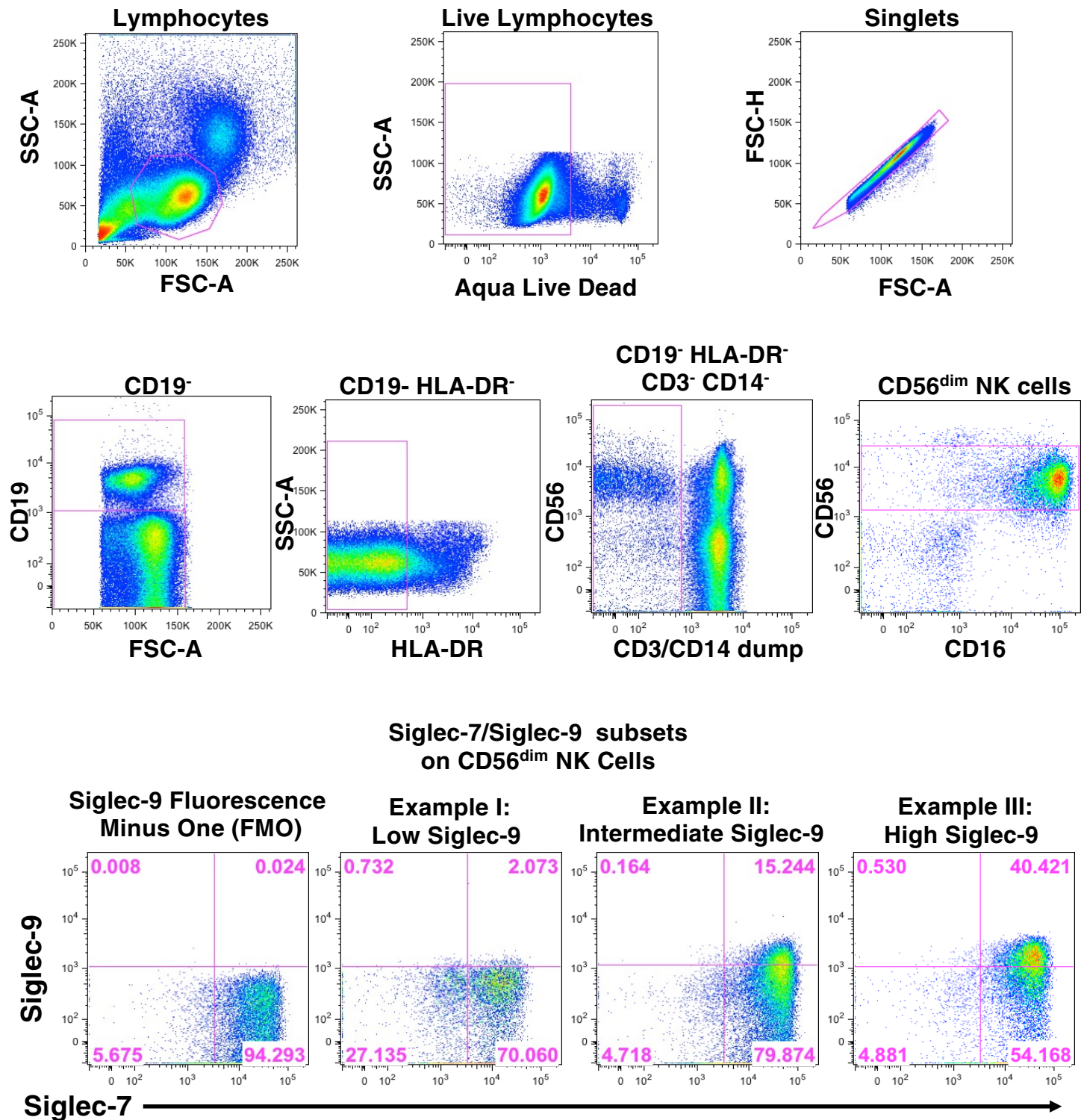

Supplement: FIG S5 [file mbio.03393-22-s0005.pdf]

Supplementary Figure 6

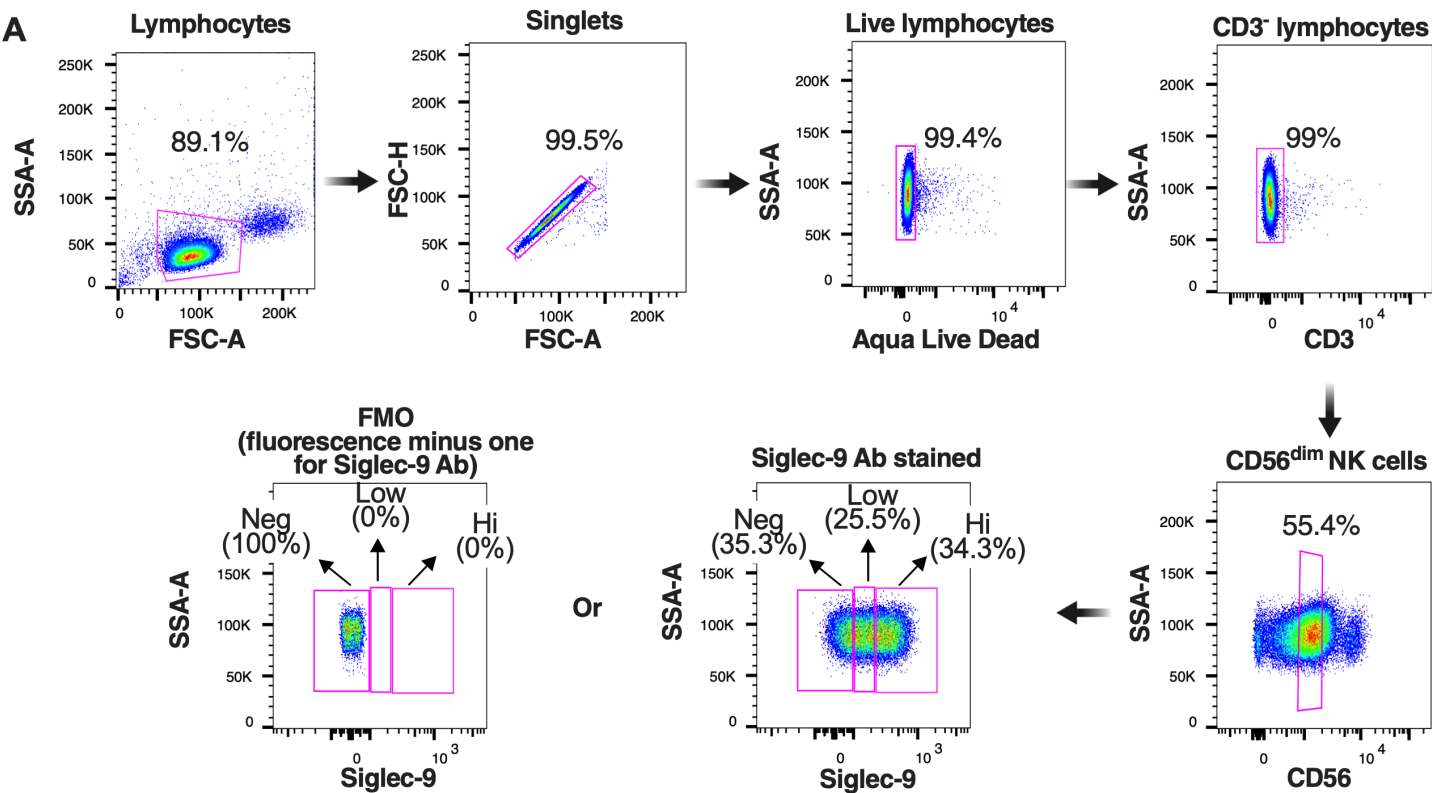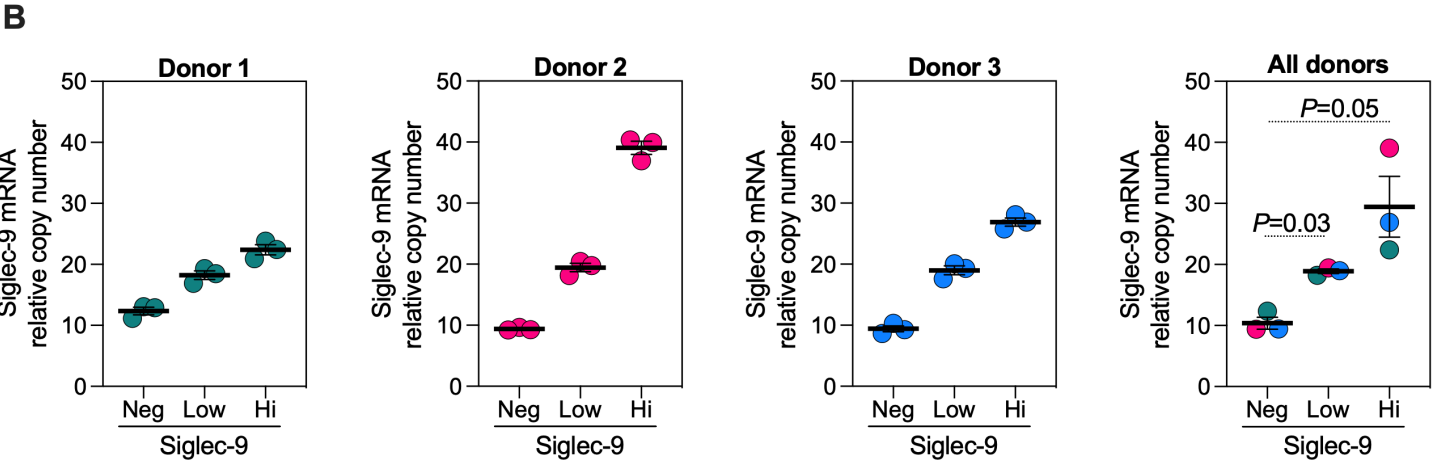

Supplement: FIG S6 [file mbio.03393-22-s0006.pdf]

## Supplementary Figure 7

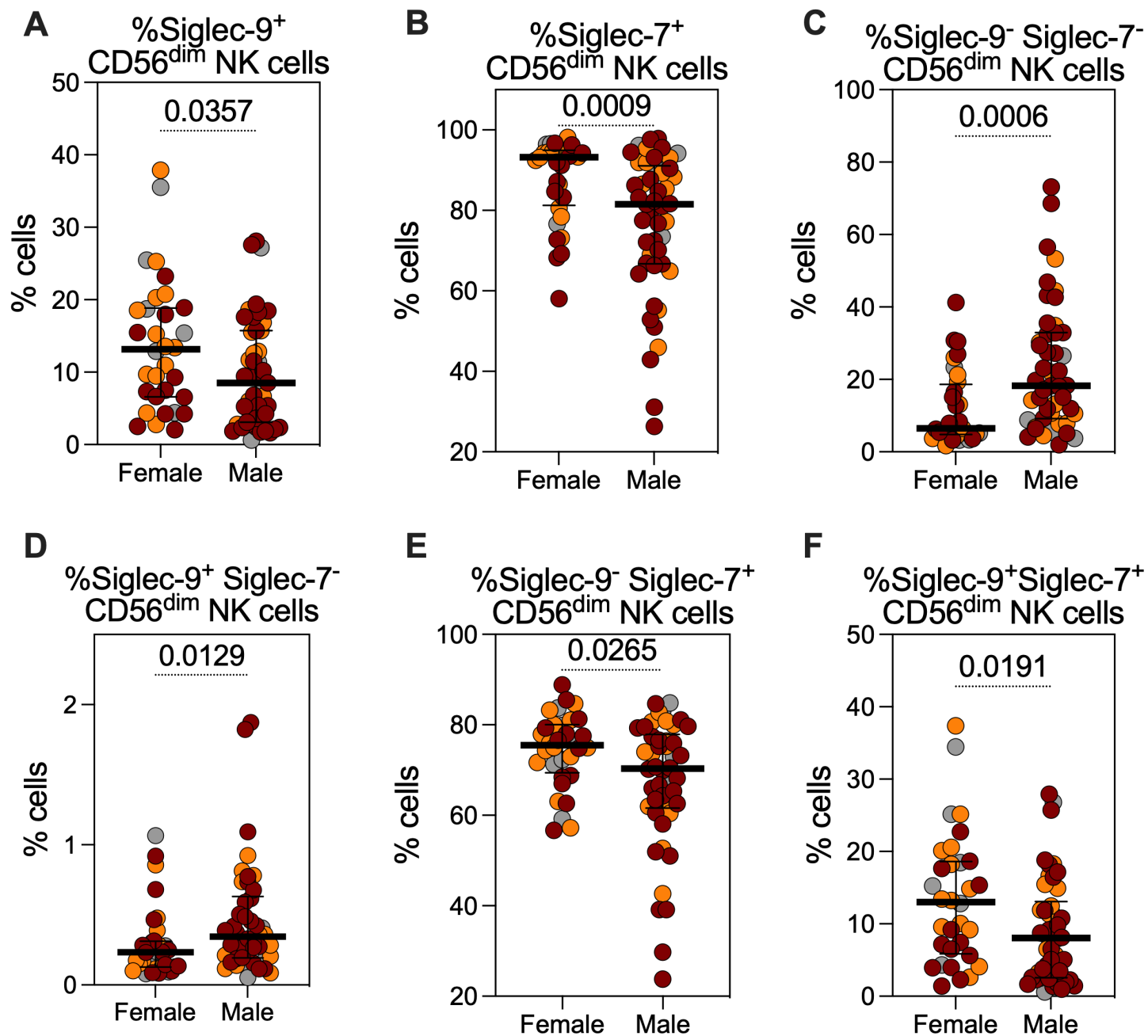

Supplement: FIG S7 [file mbio.03393-22-s0007.pdf]

Supplementary Figure 8

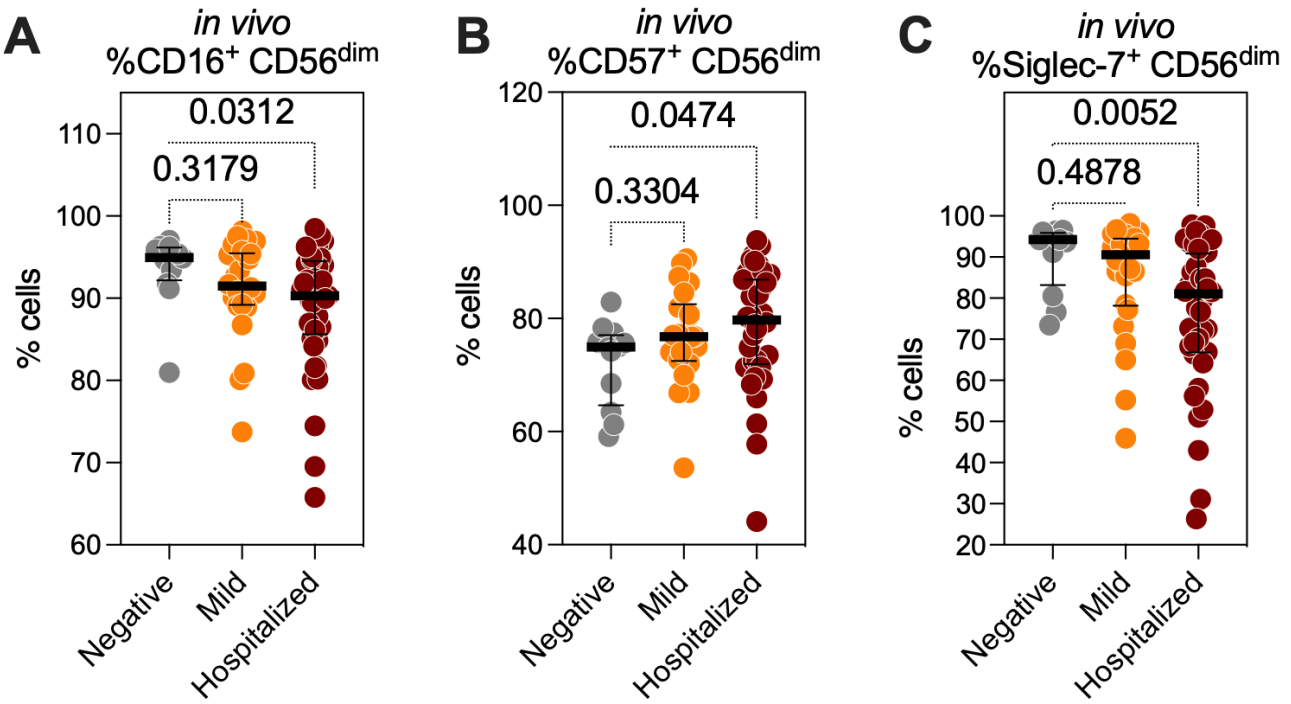

Supplement: FIG S8 [file mbio.03393-22-s0008.pdf]

**Supplementary Figure 9**

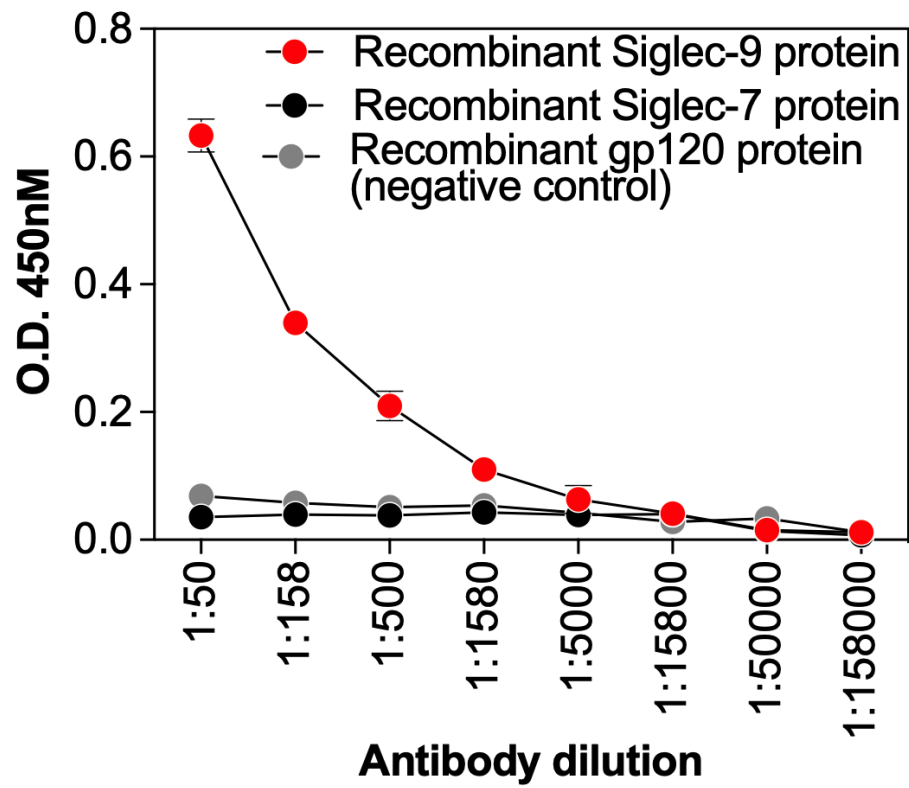

Supplement: FIG S9 [file mbio.03393-22-s0009.pdf]
